# Evaluation of molecular interactions of vaping juice components with ACE2 receptor

**DOI:** 10.1038/s41598-026-39533-0

**Published:** 2026-02-21

**Authors:** Samavath Mallawarachchi, Aayushi Nangia, Mohammad Jasim Ibrahim, Aadhil Haq, Sandun Fernando, Maria D. King

**Affiliations:** 1 Department of Biological and Agricultural Engineering, Texas A&M University, College Station, TX 77843 USA; 2 Department of Chemical Engineering, Texas A&M University, College Station, TX 77843 USA

**Keywords:** ACE2 receptor, Vape juice, Nicotine, Electronic nicotine delivery, Computational toxicology, Receptor-centric analysis, Ligand-receptor interactions, Biochemistry, Chemical biology, Chemistry, Drug discovery

## Abstract

Electronic nicotine delivery systems have recently achieved great popularity as safer alternatives to traditional tobacco. However, there is growing evidence that vaping is not harmless, with documented acute and chronic health effects. This study investigated how components of vape juice interact with human Angiotensin-converting enzyme 2 (ACE2) receptors, using molecular docking, molecular dynamics simulations, and Biolayer Interferometry (BLI). In the initial docking, menthol showed the strongest binding, followed by nicotine and capsaicin, while formaldehyde and acrolein demonstrated moderate binding to the zinc ion binding site of ACE2. Capsaicin formed the greatest number of interactions with multiple hydrogen bonds targeting the catalytic HIS374 residue. Menthol, glycerol, and propylene glycol also formed hydrogen bonds in the active site region, while nicotine formed polar interactions. In contrast, formaldehyde and acrolein, the product of glycerol and propylene glycol, did not form any significant interactions with ACE2, maybe due to their small molecule size. All vaping components formed interactions with the Zn^2+^ ion, suggesting potential implications on active site functionality. In the BLI experiments, nicotine demonstrated the most stable binding to ACE2, as evidenced by slow dissociation. The binding of menthol and capsaicin to ACE2 was less stable compared to nicotine, possibly due to their hydrophobic nature. These findings can pave the way for future studies exploring the vaping-related effects within a receptor-centric systems biology framework.

**Supplementary Information:**

The online version contains supplementary material available at 10.1038/s41598-026-39533-0.

## Introduction

The exponential rise in the global use of electronic nicotine delivery systems (ENDS), particularly among adolescents and young adults, has outpaced scientific understanding of their molecular and systemic consequences. Recent systematic reviews and meta-analyses have highlighted the global acceleration of ENDS use, estimating the prevalence of ever-use and current use among adolescents at approximately 17% and 5%, respectively. The highest prevalence of youth vaping is observed in Europe and North America. In a 2024 study on e-cigarette use among US students, flavorants and perceived harm reduction were cited as key drivers of initiation^1^. Marketed as safer alternatives to traditional tobacco, these devices generate aerosols by heating liquids containing nicotine, flavorants, solvents, and additives. However, mounting evidence indicates that vaping is far from benign, with acute and chronic effects now well documented in clinical, epidemiological, and experimental studies^2,3^.

Vape juices, or e-liquids, are primarily composed of a base mixture of propylene glycol (PG) and vegetable glycerin (VG), commonly in ratios such as 50:50 or 70:30 (VG: PG), which together account for approximately 80–95% of the total volume of the liquid. Nicotine, ranging from 0 to 30 mg/mL based on the formulation and user preference, and a diverse array of chemical flavorants, typically comprising 5–10% of the mixture, are added to enhance sensory appeal and reinforce behavioral patterns^4–7^. While PG and VG serve as the major carriers and determine vapor characteristics, the specific blend of nicotine and flavorings, including agents such as menthol, vanillin, and ethyl maltol, critically shapes the physiological and behavioral responses to vaping. Minor constituents or byproducts such as formaldehyde and acrolein may also arise due to thermal degradation, with implications for lung and vascular health^8,9^.

Previous studies have shown that the amount of nicotine delivered by e-cigarettes is much higher than the amount delivered via cigarette smoke^10,11^. As a result, aerosolized constituents may interact with neuroactive and regulatory receptors, including nicotinic acetylcholine receptors (nAChRs), angiotensin-converting enzyme 2 (ACE2), and transient receptor potential (TRP) channels^12^. It is reported that a single vaping session achieves > 80% α4β2 receptor occupancy, mimicking the neurochemical reinforcement of conventional tobacco use^13^. This convergence of sensory appeal and receptor engagement underscores the need for molecular-level investigations to clarify how individual vaping constituents interact with biologically relevant targets.

ACE2, known as the cellular entry receptor for SARS-CoV-2, has been proposed to be regulated by nAChRs. These receptors are broadly expressed throughout the central nervous system, making them highly responsive to nicotine. Upon activation, they can trigger and sustain a reward-related signaling cascade, potentially contributing to the development of nicotine addiction^14,15^. Beyond neuronal targets, evidence links nicotine and vaping-associated aldehydes with ACE2 upregulation in pulmonary epithelium, a receptor that not only modulates vascular tone but also serves as the entry point for SARS-CoV-2^16^. This modulation of the renin-angiotensin system (RAS) by vaping represents a previously underappreciated mechanism that may predispose users to respiratory infections, vascular remodeling, or hypertensive sequelae^16,17^. Compounding this effect are potent flavorants such as menthol and cinnamaldehyde, which potently activate transient receptor potential (TRP) channels, including TRPM8, TRPA1, and TRPV1, which play a central role in regulating sensory perception^18–24^.

While much of the existing literature has focused on expression-level effects on ACE2, ACE2 also contains a catalytic pocket which is capable of engaging with small molecules. Previous studies have demonstrated that small molecules can bind to the active site of ACE2 and modulate enzyme activity via hydrogen bonding, electrostatic or Zn^2+^ coordination interactions^25–28^. Therefore, direct binding of vaping components on ACE2 active site is a rational approach to understand if these substances can occupy or perturb the catalytic site of ACE2. We hypothesized that common vape juice components, including nicotine, flavorants, and degradation byproducts, directly interact with the ACE2 receptor at its catalytic binding site, and that the stability and binding characteristics of these interactions differ substantially among constituents.

This study utilizes molecular docking and molecular dynamics simulations to identify high-affinity interactions between ACE2 and vape juice components and associated conformational changes. These computational findings are further supported by experimental validation of the binding kinetics of nicotine, and the flavorants menthol and capsaicin to the ACE2 receptor using biolayer interferometry (BLI). This serves as an initial step toward quantifying receptor-specific binding constants under conditions that closely mimic physiological environments.

The majority of the existing literature on ACE2-ligand interactions have focused on viral spike protein binding or antiviral drug discovery^29–35^. Prior work evaluating vaping-related effects on ACE2 has primarily emphasized nicotine, largely in the context of expression-level regulation, leaving the molecular impact of other common vape constituents largely uncharacterized^16,30,31^. Our study analyzes an understudied aspect of ACE2 by systematically evaluating the molecular-level interactions of a broader panel of vape juice components with ACE2, using an integrated approach based on computational analysis and experimental validation. This study expands beyond previous nicotine-focused work to include flavoring agents, solvents, and byproducts. This helps to obtain a more comprehensive mechanistic understanding of how diverse vape constituents may engage the ACE2 receptor at the molecular level. These findings could contribute to changing from viewing vaping as primarily a behavioral or chemical toxicology issue to understanding its effects within a receptor-centric framework.

## Methods

### Receptor preparation

The human angiotensin-converting enzyme 2 (ACE2) crystal structure (PDB ID: 3D0G) was obtained from the Protein Data Bank (https://www.rcsb.org/), resolved at 2.80 Å by X-ray diffraction^36^. This crystal structure represents the spike protein receptor-binding domain from the 2002–2003 SARS coronavirus human strain complexed with a human-civet chimeric ACE2 receptor. This structure was selected since it represents a high-resolution structure with a clearly resolved catalytic domain including the Zn^2+^ ion and has been used in several computational studies^37–40^. The protein structure contained four polypeptide chains, A and B representing the ACE2 receptor, and E and F representing the spike protein. Since chains A and B represent identical subunits of ACE2, and the binding site is located away from the dimer interface, Chain B was used for molecular docking and molecular dynamics simulations in this study.

Optimization of the protein structure was performed by Schrödinger Protein Preparation Wizard. The preprocessing involved filling missing side chains, assigning bond orders, adding hydrogens, and removal of water molecules. Following that, the structure was subjected to hydrogen bond optimization and minimization.

### Protein structure validation

To ensure the accuracy and reliability of the ACE2 receptor structure prior to docking and simulation, multiple validation tools were employed. The PROCHECK server (https://www.ebi.ac.uk/thornton-srv/software/PROCHECK/) was used to examine the stereochemical quality of the protein by performing residue-level analysis of backbone dihedral angles^41^. The resulting Ramachandran plot provided insights into the distribution of residues within energetically favorable regions, thereby evaluating overall folding and conformational stability^42^. The processed ACE2 structure containing only Chain B was submitted to PROCHECK for quality assessment.

In addition, the structural compatibility between the three-dimensional coordinates and the one-dimensional amino acid sequence was assessed using the Verify3D server (https://www.doe-mbi.ucla.edu/verify3d/)^43^. This tool evaluated the compatibility of each residue with its surrounding environment by analyzing local structural features such as α-helices, β-sheets, and loop regions, ensuring consistency between sequence and fold.

Further validation was performed using the ProSA-web server (https://prosa.services.came.sbg.ac.at/prosa.php), which calculates residue-wise energy profiles and an overall Z-score for the model. The Z-score obtained was compared with experimentally solved protein structures of similar size within the Protein Data Bank, enabling assessment of model reliability relative to validated crystallographic entries^44^. Together, these complementary validation strategies ensured that the ACE2 structure was of sufficient quality for subsequent molecular docking and molecular dynamics simulations.

To compare this structure with other reported structures, we superposed this with a ligand-free ACE2 structure^25^, which confirmed preservation of the catalytic Zn²⁺ center and active site geometry.

### Ligand preparation

The three-dimensional (3D) structures of the target ligands which includes nicotine (PubChem CID: 89594), menthol (PubChem CID: 1254), capsaicin (PubChem CID: 1548943), formaldehyde (PubChem CID: 712), propylene glycol (PubChem CID: 1030), glycerol (PubChem CID: 753) and acrolein (the unsaturated aldehyde byproduct from glycerin and propylene glycol) (PubChem CID: 7847) were retrieved from the PubChem database (https://pubchem.ncbi.nlm.nih.gov/). MLN4760 (PubChem CID: 448281), a known ACE2 inhibitor reported to bind to Zn^2+^ binding site of ACE2^25^, was used as a positive control. Each ligand structure was optimized using the Schrödinger LigPrep tool employing OPLS4 force field.

The ligands were selected based on their prevalence in vape juice and reported ability to cause physiological impacts. Propylene glycol and glycerol are the main components of vape juice and generally comprise 80–95% of the vape juice volume. The vape juice is also reported to contain 6–30 mg/L of nicotine, and 5–10% of flavoring components such as menthol and capsaicin^4–7^. Menthol and capsaicin were picked among the flavoring constituents based on their prevalence in commercial e-liquids and their robust ability to activate TRP channels^8,45^. Capsaicin was selected as a representative vanilloid compound, a structural class that includes vanillin and related flavorants, since it is a potent TRPV1 agonist which has been extensively used to study airway nociception, cough reflexes, and inflammatory responses in respiratory system, making it a useful mechanistic probe to study vanilloid-receptor interactions^46–49^. While formaldehyde and acrolein are only present in low concentrations, they were selected due to their reported toxicological relevance^9^. This selection strategy ensured that experimental analyses focused on ligands with the highest relevance to vaping-associated health outcomes.

### Molecular docking

The components of vape juice were docked on human ACE2 receptor using Schrödinger Glide 2023. The docking was conducted at the Zn^2+^ binding site, which is also identified to bind small-molecule inhibitors, and contained a surface topology suitable for small molecule binding^25,50^. The docking grid was centered around the critical catalytic residues HIS374, HIS378, and GLU402^25^, and the grid size was maintained at 20 Å. The docked complexes were visualized using Schrödinger Maestro, and the interactions between ACE2 and each ligand were analyzed using Schrödinger Ligand Interaction Diagram. All docking scores were expressed as the average of the three strongest binding conformations at the docking site.

### Molecular dynamics simulations

Molecular dynamics (MD) simulations were carried out for each ACE2-ligand complex using the Desmond module of Schrödinger 2023 to evaluate the dynamic stability of binding interactions under physiologically relevant conditions. System preparation was carried out using the OPLS4 force field and TIP3P solvent model, with parameters automatically assigned to both protein and ligands. The simulations were conducted for 300 ns with a recording interval of 20 ps, using the NPT ensemble at 1.01325 bar and 300 K. The Nose–Hoover chain method with a 1.0 ps interval served as the thermostat, and the Martyna–Tobias–Klein method with a 2.0 ps interval was used as the barostat.

Post-simulation analyses were carried out using the Simulation Interaction Diagram tool in Maestro, which provided quantitative assessments of root mean square deviation (RMSD), root mean square fluctuation (RMSF), solvent accessible surface area (SASA), and ligand-protein interaction persistence across the simulation trajectories. RMSD values were calculated using the initial conformation as the reference. To further quantify binding affinities, MM-GBSA (Molecular Mechanics Generalized Born Surface Area) free energy calculations were performed on representative snapshots from the equilibrated trajectories using the thermal_mmgbsa.py script. Energy values were calculated based on 250 frames during the last 50 ns of the simulation. These calculations combined molecular mechanics energies with solvation effects to yield the relative binding free energies (ΔGbind) for each ligand-ACE2 complex. Since the MM-GBSA energy calculation does not incorporate entropic contributions, entropic contributions to the binding were determined using the interaction entropy (TΔS) approach introduced by Duan et al^51^. The following equation was used to calculate interaction entropy, and the ΔE values were calculated based on 250 frames during the last 50 ns of the simulation^51,52^.

$$\:T\Delta\:S\:=\:-RT\:ln<{e}^{\left(\frac{{\Delta\:E}_{int}\:-\:<{\Delta\:E}_{int}>}{RT}\right)}>\mathrm{w}\mathrm{h}\mathrm{e}\mathrm{r}\mathrm{e}\:\Delta\mathrm{E}_{\mathrm{i}\mathrm{n}\mathrm{t}}\hspace{0.17em}=\hspace{0.17em}\Delta\mathrm{E}_{\mathrm{c}\mathrm{o}\mathrm{u}\mathrm{l}\mathrm{o}\mathrm{m}\mathrm{b}}\hspace{0.17em}+\hspace{0.17em}\Delta\mathrm{E}_{\mathrm{v}\mathrm{a}\mathrm{n}\:\mathrm{d}\mathrm{e}\mathrm{r}\:\mathrm{W}\mathrm{a}\mathrm{a}\mathrm{l}\mathrm{s}}$$

This integrated approach, combining molecular docking, molecular dynamics simulations, and MM-GBSA free energy analysis, follows a commonly used workflow in characterizing ligand binding to ACE2 and other receptors^53–57^.

### Principal component analysis

Principal component analysis (PCA) of MD trajectories was conducted on the α-Carbon atoms of the ACE2 protein. Prior to the simulation, the trajectory was aligned to the first frame to remove global translational and rotational movements, and the time-averaged mean structure was subtracted from all the frames. PCA was computed using Singular Value Decomposition (SVD) of the coordinate matrix, using the Python script provided in the supplementary data. The variance of each component was calculated using the squared singular values and normalized to determine the percentage of total variance explained by each component. Projections of each frame along the first two principal components were computed and plotted to obtain a visual representation of the most significant motions.

Free energy landscapes (FEL) for each ACE2-ligand complex were constructed by mapping the relative free energy across conformational space as a function of the first two principal components (PC1 and PC2). The probability distribution along these two components was calculated by normalizing the two-dimensional histogram of PC1 and PC2 projections, and the free energy (G) corresponding to each conformational state was calculated using the following equation^58^.

G = -k_B_T ln P, where k_B_ is Boltzmann constant, T is absolute temperature and P represents the probability distribution of the system along the two principal components. The resulting free energy surfaces were shifted such that the global minimum corresponded to zero free energy.

### Biolayer interferometry

Biolayer interferometry (BLI) was conducted using the Sartorius Octet R4 BLI Label-Free Detection system (Sartorius, Ann Arbor, MI). Aminopropylsilane (APS, Sartorius) biosensors were hydrated for 10 min in a pH 7.4 phosphate buffered saline (PBS). The hydrated biosensors were initially equilibrated in PBS for 60 s to establish a baseline, then the human ACE2 protein (Sino Biological US Inc., Houston, TX) was loaded on the biosensors for 450 s at a concentration of 5 µg/mL. Nicotine, menthol, capsaicin (a representative of vanilloids), and acrolein were purchased from WVR Avantor (Radnor, PA). This was followed by a second baseline step of 60 s, and association and dissociation of 300 s each. For each compound, association was done at the concentrations of 10, 5, 2.5, 1.25, 0.625, and 0.3125 mM, with zero concentration as the reference. Lower concentrations were used for capsaicin due to solubility constraints. A parallel set of reference biosensors not loaded with ACE2 was used to mitigate the impact of non-specific binding, and the kinetic parameters were calculated after subtracting the readings of the reference sensors. All solutions were prepared in pH 7.4 PBS, which mimics the physiological pH and ionic strength. Due to the low solubility of menthol and capsaicin, 1% DMSO was used in all menthol and capsaicin solutions, in both reference and testing assays. This DMSO concentration is within the acceptable range for BLI and its consistent inclusion in both reference and testing assays minimizes its impacts on kinetic parameters^59,60^.

The binding kinetic parameters were calculated using Octet Analysis Studio software, using global fitting of association and dissociation curves to 1:1 binding model.

## Results and discussion

### Protein structure validation

**Fig. 1:**
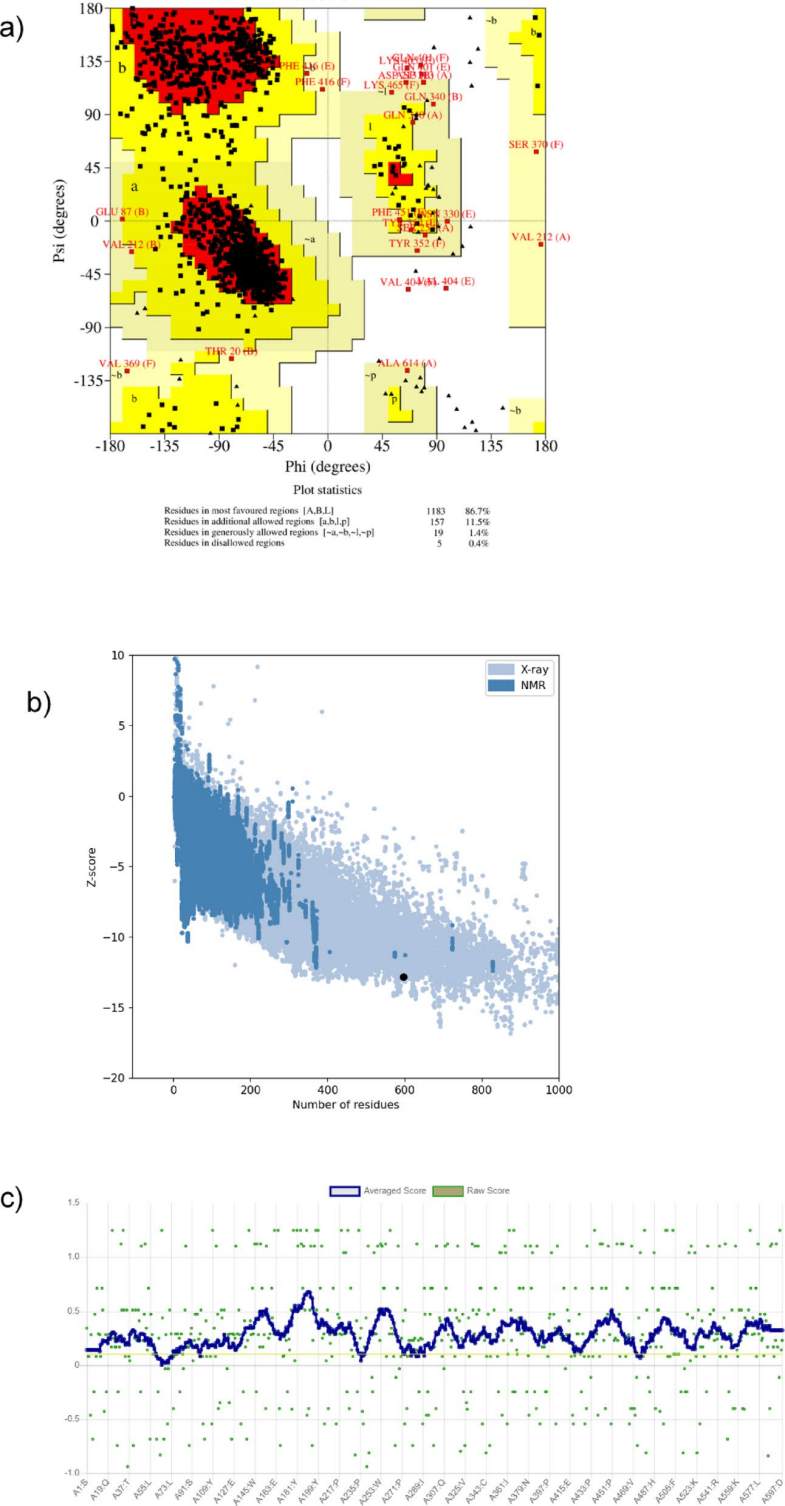
**(a)** PROCHECK Ramachandran plot of the 3D0G protein illustrating the ψ and φ dihedral angles of its amino acid residues. Amino acids residing in the most favored regions are depicted in red, while those in additional allowed regions are highlighted in bright yellow. **(b)** ProSA-web validation of the ACE2 structure. The Z-score of − 12.82 (indicated by the black dot) falls within the range characteristic of experimentally determined protein structures of comparable size. **(c)** Verify3D analysis of the ACE2 model. A total of 94.2% of residues exhibited acceptable 3D–1D compatibility scores, with at least 80% of residues scoring ≥ 0.1. Overall, this analysis confirms the stereochemical quality of the protein structure.

The stereochemical quality of the refined ACE2 protein was assessed using the Ramachandran plot generated by PROCHECK, z-score analysis with ProSA, and 3D-1D analysis by Verify 3D, which are presented in Fig. 1. The Ramachandran plot (Fig. 1a) showed that 86.7% of the residues were located in the most favored regions, indicating strong structural regularity and stability of the model. Further 11.5% residues were present in additionally allowed regions, and 1.4% fell within generously allowed regions, both of which are considered acceptable for high-quality protein structures. Only 0.4% (5 residues) were located in disallowed regions, suggesting minimal stereochemical deviations and confirming the reliability of the structural refinement^41^.

Further evaluation with the ProSA-web server (Fig. 1b) yielded a Z-score of − 12.82, which lies within the range of experimentally validated crystal structures of comparable size, confirming the overall accuracy and stability of the ACE2 model^44^. Complementary analysis with the Verify3D server demonstrated that 94.2% of residues achieved acceptable 3D–1D compatibility scores, with at least 80% of residues scoring ≥ 0.1 (Fig. 1c), confirming consistency between the amino acid sequence and the modeled three-dimensional environment. Together, these independent validation metrics confirm that the refined ACE2 structure is of high stereochemical quality and suitable for downstream molecular docking and molecular dynamics simulations with vape-derived ligands.

### Molecular docking

**Fig. 2:**
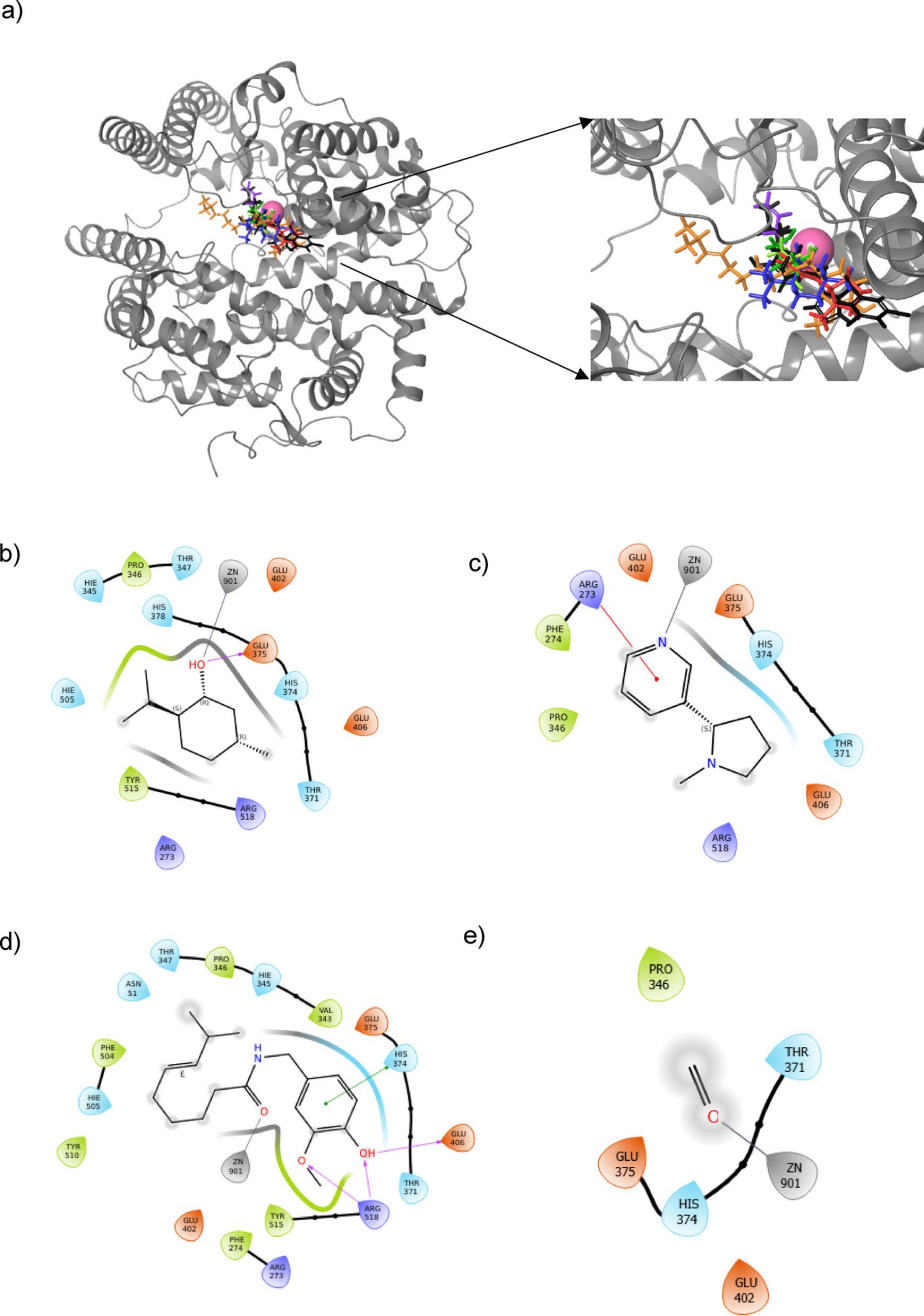

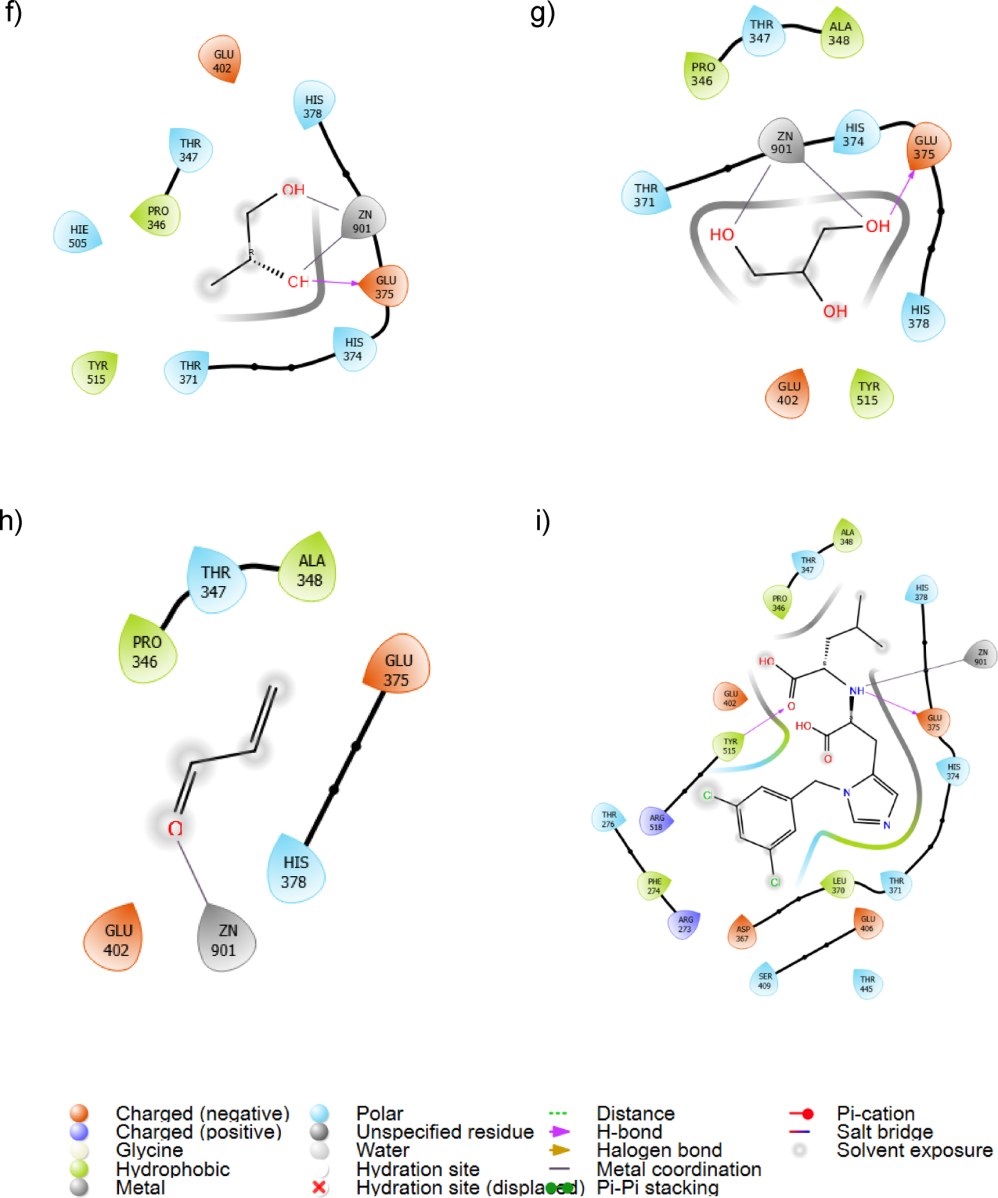
**(a)** Docking conformations of menthol (blue), nicotine (red), capsaicin (orange), formaldehyde (yellow), propylene glycol (light green), glycerol (green), acrolein (purple), and MLN4760 (black) on the Zn^2+^ binding site of ACE2. Zn^2+^ is depicted as a pink sphere. Interactions of **(b)** menthol, **(c)** nicotine, **(d)** capsaicin, **(e)** formaldehyde, **(f)** propylene glycol, **(g)** glycerol, **(h)** acrolein, and **(i)** MLN4760 with ACE2 at the active site.

Seven components of vape juice: nicotine, menthol, capsaicin (a representative of vanilloids), formaldehyde, glycerol, propylene glycol, and acrolein were docked on the active site of the human ACE2 receptor, with MLN4760 as a positive control. The docking scores of the vape juice components at the Zn^2+^ binding site of the ACE2 receptor are given in Table 1, and the best binding conformations of those components and their interactions with ACE2 are visualized in Fig. 2.

As observed in Fig. 2a, all molecules bound to the same region in the Zn^2+^ binding pocket of the ACE2 receptor. The close proximity of these molecules suggests their potential to interact with the Zn^2+^ ion and critical catalytic residues.

Analysis of docking scores showed that menthol exhibited the strongest binding to ACE2 among vape juice components, with an average docking score of −4.410 kcal/mol, followed by nicotine, capsaicin, and formaldehyde, which demonstrated moderate binding. The docking score of menthol was comparable to the docking score of positive control MLN4760, while nicotine and capsaicin demonstrated slightly weaker binding.

Pairwise RMSD values between the three strongest binding conformations were analyzed to verify the reproducibility of binding (Table 1). It can be observed that all ligands showed average RMSD values less than 2 Å, indicating good convergence and reproducibility^61^. The binding reproducibility was also verified by visual inspection of the best binding conformations of each ligand, which revealed the top-ranked docking poses for each ligand clustered within the same region of the ACE2 catalytic pocket, as depicted in Supplementary Figure S1.

Analysis of interactions (Fig. 2b-h) revealed that capsaicin formed the greatest number of interactions with ACE2, with multiple hydrogen bonds and a π-π stacking interaction with catalytic residue HIS374. Menthol, glycerol, and propylene glycol also formed hydrogen bonds with GLU375 in the active site region, while nicotine formed polar interactions with the catalytic region and a pi-cation interaction with ARG273. MLN4760 formed hydrogen bonds with GLU375 and TYR515, the residues reported to be in close proximity to MLN4760 binding site in experimental studies^25^. Overall, all these molecules interacted with residues in the HIS374-HIS378 range, which contains key residues essential for the catalytic activity of ACE2^25^. In comparison, formaldehyde and acrolein did not form any significant interactions with ACE2, which may be attributed to their small molecule size.

Notably, all molecules exhibited close proximity to the Zn^2+^ ion at the active site and formed interactions with the Zn^2+^ ion. The distance between Zn^2+^ and interacting atoms in the ligands, which ranged from 2.02 to 2.23 Å (Table S1), was consistent with Zn^2+^ coordination bond length reported in the literature^62^. This suggests potential metal coordination with the Zn^2+^ ion. However, further quantum mechanics analysis is necessary for definitive validation of Zn^2+^ coordination.

Overall, molecular docking revealed that menthol, nicotine, and capsaicin showed moderate initial binding to the active site of ACE2. Therefore, these three compounds were selected for further analysis based on docking results, while formaldehyde and acrolein were also selected due to their reported carcinogenic and mutagenic properties^63,64^.

**Table 1:** Docking scores of vape juice components on the active site of the ACE2 receptor. All Docking scores are reported as the average ± standard deviation based on the three strongest binding conformations.

Compound	Average docking score at the Zn^2+^ binding site (kcal/mol)	Average RMSD among top three conformations (Å)
Menthol	−4.410 ± 0.117	0.3274
Nicotine	−3.718 ± 0.050	1.4018
Capsaicin	−3.520 ± 0.407	1.9909
Formaldehyde	−3.337 ± 0.078	0.0005
Propylene glycol	−2.796 ± 0.067	0.4348
Glycerol	−2.623 ± 0.087	0.8420
Acrolein	−0.820 ± 0.064	0.4315
MLN4760 (control)	−4.464 ± 0.112	1.6036

### Molecular dynamics simulations

**Fig. 3:**
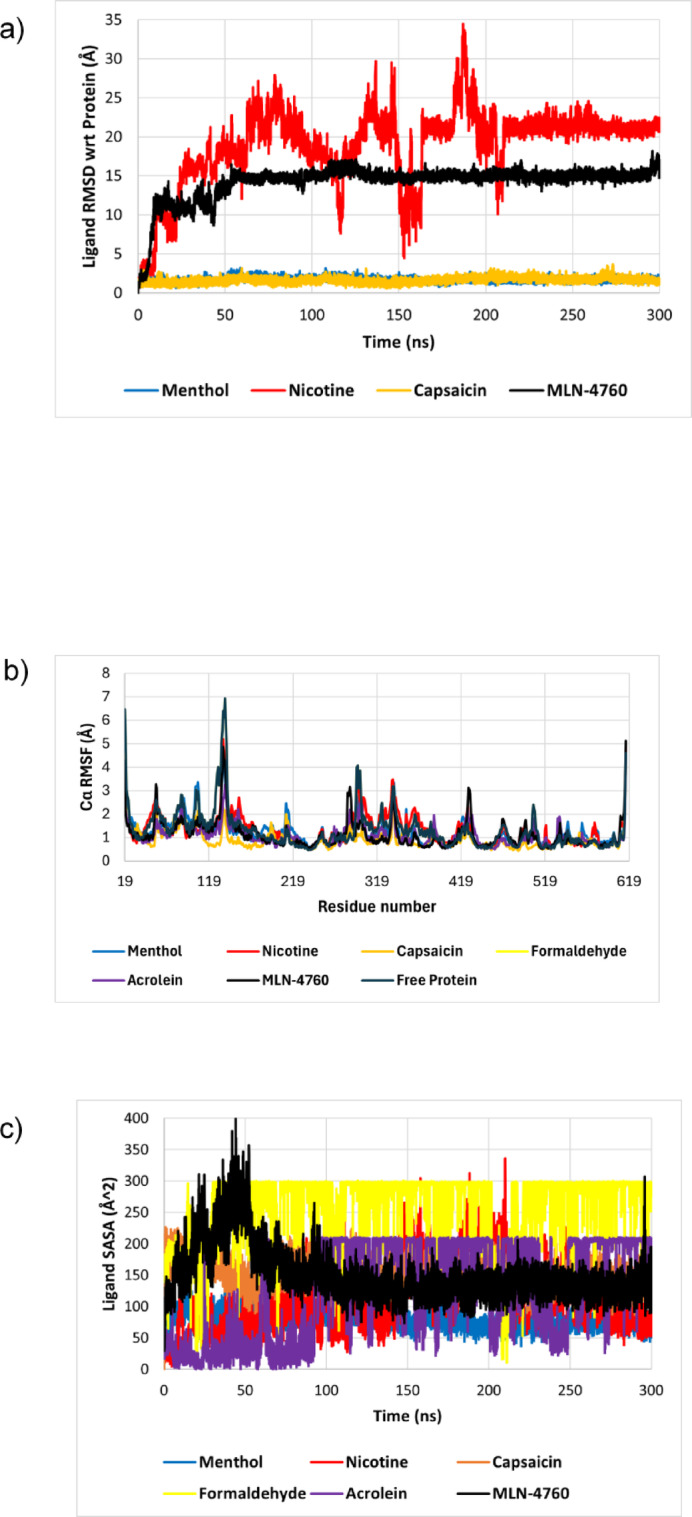
**(a)** Variation of ligand RMSD with respect to protein over the simulation duration for menthol, nicotine, and capsaicin, **(b)** Protein RMSF plots for ACE2 complexed with different vape juice components, and **(c)** Variation of SASA of vape juice components over simulation duration. Menthol and capsaicin demonstrate stable binding to the active site.

**Fig. 4:**
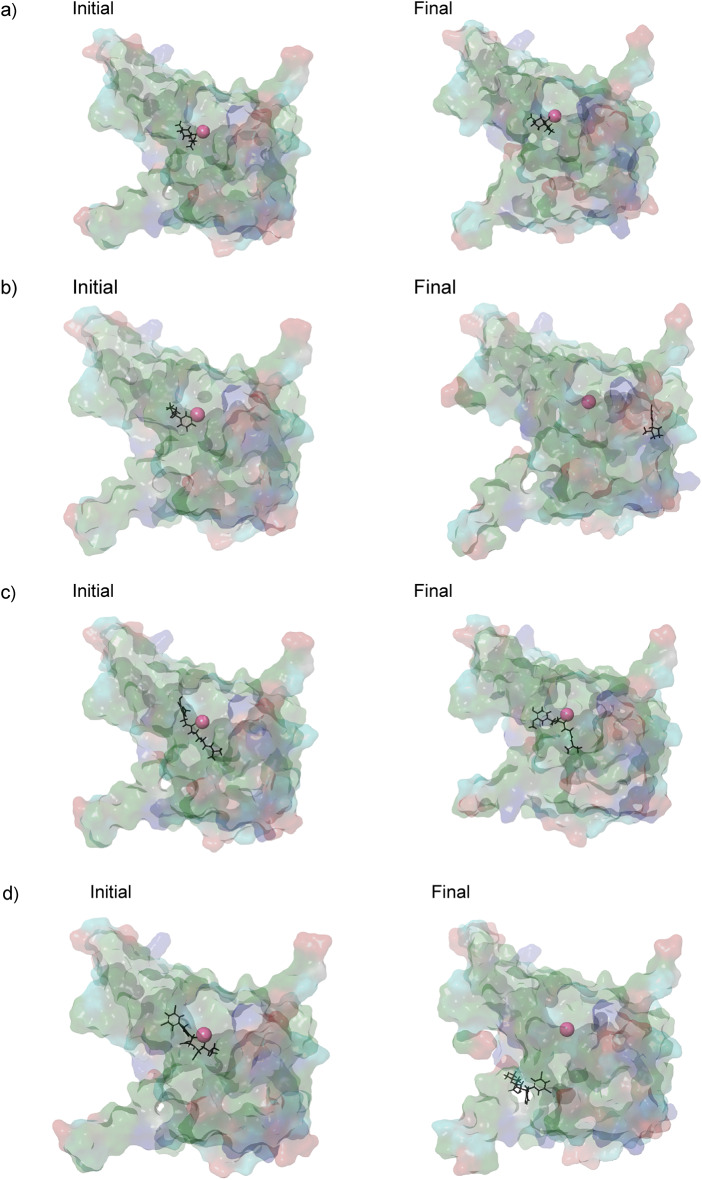
Initial and final frames of the MD simulations for **(a)** menthol, **(b)** nicotine, **(c)** capsaicin and **(d)** MLN4760. The ligand is shown in black and Zn^2+^ ion is depicted as a pink sphere. Hydrophobic (green), polar (cyan), positively charged (blue) and negatively charged (red) residues interact with the vape juice components in the initial and final frames.

Molecular dynamics (MD) simulations were performed to assess the stability of vape juice components bound to ACE2. The binding stability of the ligands was analyzed using ligand RMSD and RMSF, while protein RMSF was used to evaluate residue-wise fluctuations of the receptor. SASA represents the surface area that can interact with the solvent. Results of the RMSD, RMSF, and SASA analysis are presented in Table 2; Fig. 3, initial and final conformations of the stable binding ligands at the binding site are illustrated in Fig. 4, and the trajectory videos are included in supplementary data.

Among the five tested compounds, menthol and capsaicin exhibited the highest stability when complexed with ACE2, as shown by their low ligand RMSD and RMSF values, and stable SASA (Table 2). Their RMSD values are comparable to those typically reported for stable protein–ligand complexes in the literature^65,66^. They also achieved relative stability within the first 20 ns of the simulations and displayed only minor fluctuations after that (Fig. 3a and c). While capsaicin demonstrated relatively higher SASA, it can be attributed to the presence of the hydrophobic tail, and minimal fluctuations in SASA indicate stable binding. This was also consistent with their trajectory behavior, which demonstrated that they remained bound to their initial binding sites throughout the simulation (Fig. 4 and Supplementary data).

In contrast, nicotine showed significantly higher ligand RMSD and RMSF, indicating significant deviation from the initial conformation. However, inspection of the trajectory revealed that while nicotine deviated from the initial binding pose, it relocated to an alternative position within the binding pocket after around 30 ns and demonstrated relative stability at the relocated position after some initial fluctuations (Fig. 4b and supplementary data). This behavior is consistent with its RMSD plot, which shows a rapid increase in RMSD within 30 ns, followed by some fluctuations before stabilizing at an RMSD of around 20 Å. This is further corroborated by the SASA plot, where the SASA initially increases, followed by a decrease during the last 100 ns of the simulation. Since a reduction in SASA is indicative of stable binding^67^, the reduced and stable SASA during the last 50 ns indicates that nicotine remains stably bound at the new site. Therefore, the higher mean RMSD of nicotine can be interpreted as migration into a more stable binding location rather than complete dissociation. MLN4760 also showed similar behavior and relocated to an alternative location within the binding pocket (Fig. 4d).

The smaller molecules, acrolein and formaldehyde, exhibited poor stability. As observed in their trajectory videos, they moved out of the catalytic pocket within a short time. This also agrees with their high ligand RMSD and RMSF values (Table 2), and large fluctuations in SASA (Fig. 4c), which indicate unstable binding. This suggests that formaldehyde and acrolein are unlikely to form stable interactions with ACE2.

Analysis of protein RMSF demonstrated that the protein RMSF values ranged between 0.92 and 1.31 Å for all complexes, which is close to the free-protein RMSF of 1.31 Å. The RMSF profiles of ACE2 in complex with the different ligands also closely resembled that of the free protein (Fig. 3b). This indicates that the overall structural stability or residue-wise flexibility of ACE2 has not been significantly perturbed by the binding of any of these ligands.

**Table 2:** Average ligand RMSD with respect to protein, average ligand and protein RMSF, and average ligand SASA during the MD simulation.

Compound	Average Ligand RMSD wrt protein (Å)	Average Ligand RMSF (Å)	Average Protein RMSF (Å)	Average Ligand SASA (Å^2^)
Menthol	1.717 ± 0.294	0.864 ± 0.114	1.252 ± 0.603	80.67 ± 13.43
Nicotine	18.978 ± 4.984	10.990 ± 0.273	1.315 ± 0.667	110.45 ± 47.06
Capsaicin	1.664 ± 0.358	1.092 ± 0.282	0.921 ± 0.403	154.77 ± 21.95
Formaldehyde	79.078 ± 24.498	50.764 ± 0.238	1.313 ± 0.859	257.32 ± 58.55
Acrolein	33.417 ± 15.580	36.612 ± 0.613	1.093 ± 0.465	125.01 ± 80.43
MLN4760 (control)	14.239 ± 2.341	3.945 ± 0.708	1.134 ± 0.602	150.11 ± 42.19

RMSD and RMSF of catalytic site residues (HIS374, HIS378, and HIS402) were evaluated to determine how the ligand binds affect the catalytic site specifically and are presented in Supplementary Figure S2. MLN4760 and Capsaicin caused a statistically significant reduction in global protein RMSD, indicating stabilization of the overall protein structure^68^. While a reduction in mean RMSF could be observed in HIS374 and HIS378 residues, it was not statistically significant. In contrast, nicotine led to an increase in both mean RMSD and RMSF at the catalytic site. However, the increase in RMSF was not statistically significant and is thus insufficient to come to any conclusions on active site impact. For menthol, RMSD has increased for catalytic site residues, while RMSF has not demonstrated a statistically significant change.

**Fig. 5:**
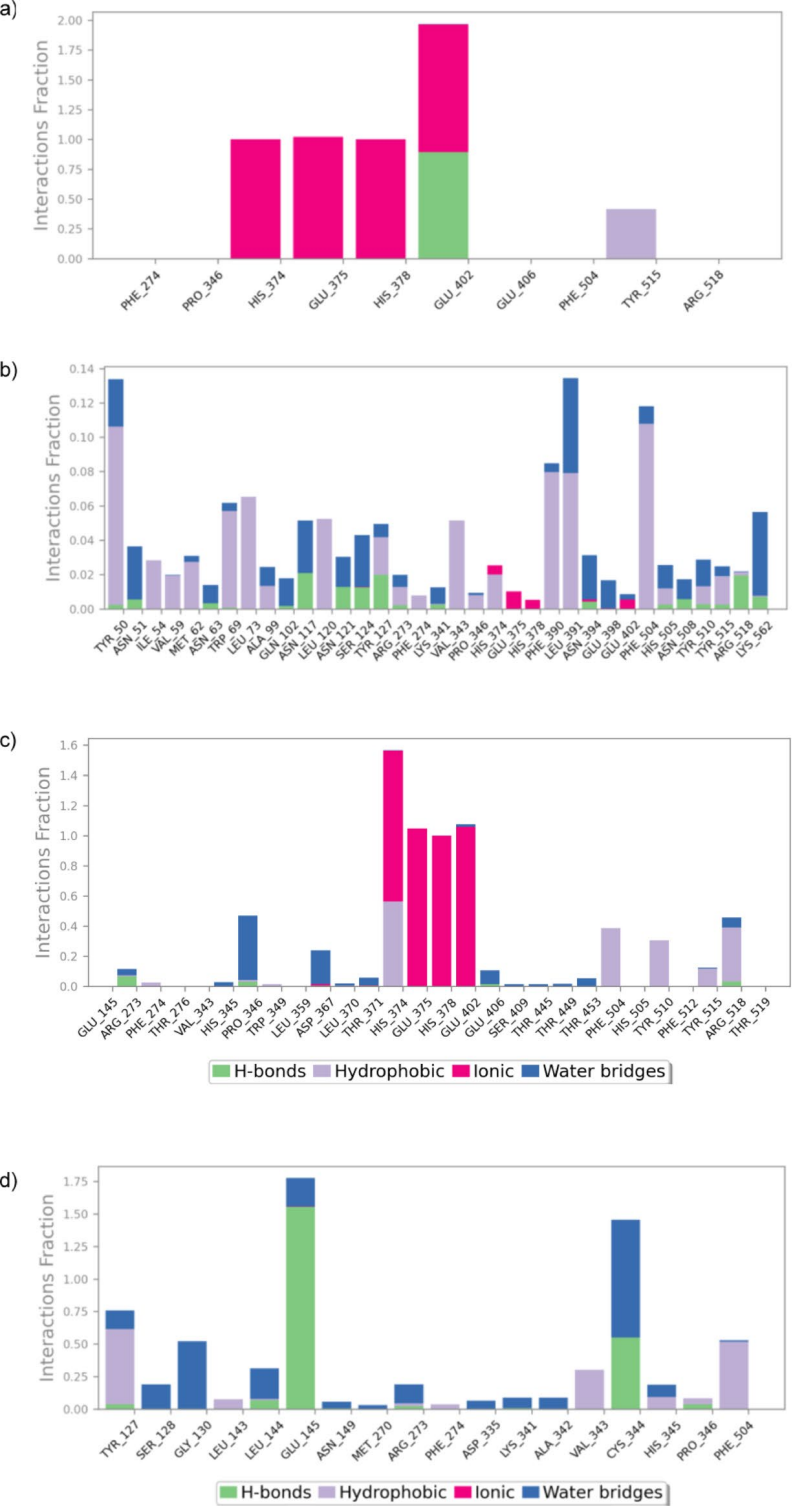
Interaction fractions of **(a)** menthol, **(b)** nicotine, **(c)** capsaicin, and **(d)** MLN4760 with different residues in ACE2. Interaction fraction is defined as the proportion of simulation time during which a specific interaction is maintained. Interaction fractions greater than 1 mean multiple interactions of the same type.

Protein ligand contacts were analyzed to elucidate how these three components interacted with ACE2 during the simulation. Both menthol and capsaicin demonstrated highly stable interactions with the catalytic residues HIS 374, HIS 378, and GLU 402, maintaining interaction fractions greater than 1 with all these residues (Fig. 5a and c). This is consistent with the trajectory videos, which showed that these two compounds remained bound to their initial binding location. Ionic interactions were predominant for these two compounds, and capsaicin also demonstrated stable hydrophobic interactions with multiple residues. In contrast, nicotine and MLN4760 formed interactions with a larger number of residues with a low interaction fraction (Fig. 5b and d). This behavior can be attributed to the deviation of nicotine and MLN4760 from their initial binding site.

Overall, the results of our MD simulations are consistent with previous computational studies examining small molecule interactions with ACE2. Recent computational studies have reported that different types of small molecules can form and maintain stable interactions with the ACE2 catalytic domain^35,50^. The binding patterns observed for menthol, nicotine, and capsaicin in our simulations are consistent with these reports and support the plausibility of vape constituents engaging the active site in a comparable manner.

### Trajectory MM-GBSA analysis

Trajectory-based MM-GBSA calculations were performed to estimate the binding free energies of the vape juice components with ACE2. This analysis provides a comprehensive overview of the binding by decomposing the binding free energy into van der Waals, electrostatic, lipophilic, solvation, and ligand strain contributions, which are presented in Table 3. MLN4760 demonstrated a highly negative binding energy of −68.761 ± 7.151 kcal/mol, indicating very strong affinity when compared to the binding energies typically reported for small molecule interactions with ACE2 and other receptors^69–72^. Among the vape juice components, nicotine showed the most favorable binding free energy (−25.061 ± 4.884 kcal/mol), indicating a moderate affinity to ACE2, primarily due to van der Waals and lipophilic interactions. This indicates that despite the relocation from the initial binding site, nicotine has maintained stable binding to the ACE2 receptor.

Menthol and capsaicin demonstrated less favorable binding, with menthol exhibiting positive binding free energy, while capsaicin showed a slightly negative ΔG bind value. This indicates that the binding of menthol and capsaicin to ACE2 is not thermodynamically favorable in modeled aqueous conditions. However, it can be observed that both menthol and capsaicin showed significantly negative electrostatic, lipophilic, and van der Waals contributions, and their high ΔG bind can be mainly attributed to high ΔG solvation values. The highly positive ΔG solvation values mean that the energy released during solvation of the complex is outweighed by the energy required for the desolvation of the receptor and ligand. Both menthol and capsaicin are hydrophobic^73,74^, and their binding to the ACE2 receptor can weaken the interactions between the receptor and surrounding water molecules, which can lead to high ΔG solvation values. Therefore, the positive binding free energies of menthol and capsaicin primarily reflect solvent-driven effects^75^. Additionally, since both ligands are highly hydrophobic and the MM‑GBSA protocol relies on an implicit solvent model with limited conformational sampling, the high ΔG solvation values may reflect an overestimation of desolvation penalties^76^. Therefore, the absolute ΔG bind values for these two ligands should be interpreted as semi‑quantitative trends rather than precise thermodynamic predictions. Especially, when these compounds are delivered in aerosolized form during vaping, they interact with the lipid-rich membranes in the respiratory tract, creating a substantially different local solvent environment than that represented in our implicit water model. Therefore, the binding energetics of menthol and capsaicin under aqueous conditions cannot be extrapolated into the physiological environment, where the proximity to hydrophobic membrane surfaces may have a substantial impact on binding energetics.

All three molecules demonstrated ligand strain energies comparable to stable interactions reported in literature, suggesting that they can easily adjust to stable conformations. Overall trajectory MM-GBSA analysis indicates that nicotine demonstrated the most thermodynamically favorable binding, while menthol and capsaicin showed strong electrostatic, lipophilic, and van der Waals contributions.

**Table 3:** MM-GBSA binding free energy components (kcal/mol) for vape chemical–ACE2 complexes. Values are reported as averages over the last 50 Ns of the MD trajectory.

Molecule	ΔG Bind	ΔG Coulomb	ΔG Covalent	ΔG Hbond	ΔG Lipophilic	ΔG Solvation	ΔG Van der Waals	Ligand Strain
Menthol	10.998 ± 2.273	−11.714 ± 1.807	−0.106 ± 0.346	−0.494 ± 0.082	−6.862 ± 0.456	51.038 ± 2.224	−20.862 ± 0.857	0.297 ± 0.428
Nicotine	−25.061 ± 4.884	−3.071 ± 1.811	0.385 ± 0.457	−0.039 ± 0.120	−12.397 ± 1.829	12.521 ± 2.355	−21.361 ± 2.934	0.626 ± 0.530
Capsaicin	−3.682 ± 3.039	−14.593 ± 2.434	1.788 ± 2.417	−0.105 ± 0.135	−14.333 ± 1.188	66.832 ± 4.073	−40.439 ± 2.116	3.940 ± 0.772
MLN4760 (control)	−68.761 ± 7.151	−20.098 ± 5.403	0.287 ± 1.447	−1.717 ± 0.419	−21.881 ± 1.797	25.263 ± 2.857	−48.583 ± 3.025	4.042 ± 1.626

Interaction entropy calculations (Table S2) revealed that the binding of all ligands is associated with an entropic penalty, which agrees with the reduction in configurational freedom upon binding. MLN4760 demonstrated the highest entropy reduction (−21.210 kcal/mol), which is indicative of tight binding that restricts conformational freedom^77^. The vape compounds exhibited entropy changes ranging from − 4 to −8 kcal/mol, indicating that their binding is associated with less conformational restrictions compared to MLN4760. As observed in Table S2, the overall ranking of free energies remained unchanged, and the binding of menthol and capsaicin remained thermodynamically unfavorable even after incorporating entropy correction.

### Principal component analysis

PCA was conducted on the Cα coordinates to further study the motion of ACE2 during the simulation. The fractions of variance explained by the top five principal components for ACE2 under different ligand-bound states are given in Table 4. For the free protein, the first three principal components (PCs) all contributed to more than 17% variance each, cumulatively accounting for 69% of the total variance. Nicotine has increased the contribution of the first PC, while reducing the contributions of other PCs, indicating that the motions of nicotine-bound ACE2 happen over a singular, dominant pathway. In menthol and MLN4760, in contrast, capsaicin-bound ACE demonstrated the lowest contribution by PC1 and greater dispersion of variance across multiple PCs, which is consistent with the reduction in global protein RMSD. While menthol and MLN4760 caused some dispersion of the variance, a dominant PC could still be observed for these complexes.

**Table 4:** Variance contributions of the first five principal components for ACE2 when complexed with ligands.

Molecule	Variance contribution (%)				
	PC1	PC2	PC3	PC4	PC5
Free protein	30.19	21.69	17.35	8.56	3.00
Menthol	20.44	13.92	4.47	3.84	3.18
Nicotine	33.12	5.01	4.52	3.64	2.91
Capsaicin	11.86	8.06	5.70	3.68	2.86
MLN4760	21.34	11.39	5.04	3.52	3.28

**Fig. 6:**
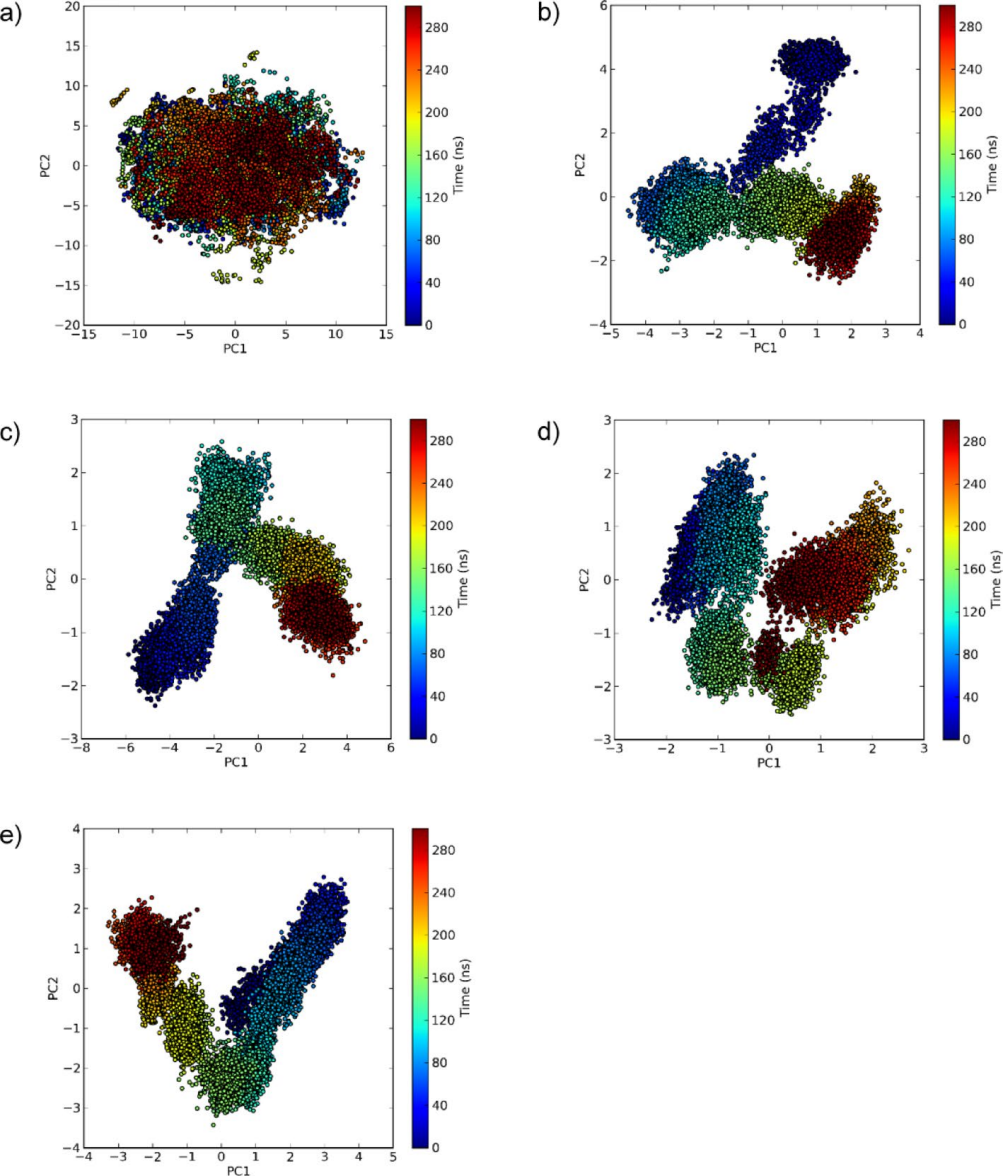
PC1 vs. PC2 projections for **(a)** free ACE protein, and ACE2 complexed with **(b)** menthol, **(c)** nicotine, **(d)** capsaicin, and **(e)** MLN4760, with frames colored based on time to illustrate conformational progression.

The PC1–PC2 projections reveal distinct dynamical behaviors of ACE2 in the free and ligand-bound states, as illustrated in Fig. 6. As observed in Fig. 6a, the free ACE2 protein is dispersed across a broad conformational space, with no distinct clustering based on time. However, when complexed with the ligands, the protein displays sequential, time-dependent clustering (from blue to red), the protein gradually transitions into distinct conformational substates over the course of the simulation. Overall, this suggests that binding of all ligands causes a contraction of conformational space compared to the free protein^78–80^.

Free energy landscapes which are presented in Supplementary Figure S3, also confirm the clear distinction in the conformational sampling between the apo-protein and the ligand-bound states. The free protein explores a broad energy landscape, which agrees with the PCA results. FEL of ACE2-menthol complex exhibits a well-defined energy minimum, consistent with its low RMSD. In contrast, the nicotine-bound complex exhibits multiple minima of comparable depth distributed across conformational space, which reflects relocation into a more stable site within the binding pocket.

### Biolayer interferometry

**Fig. 7:**
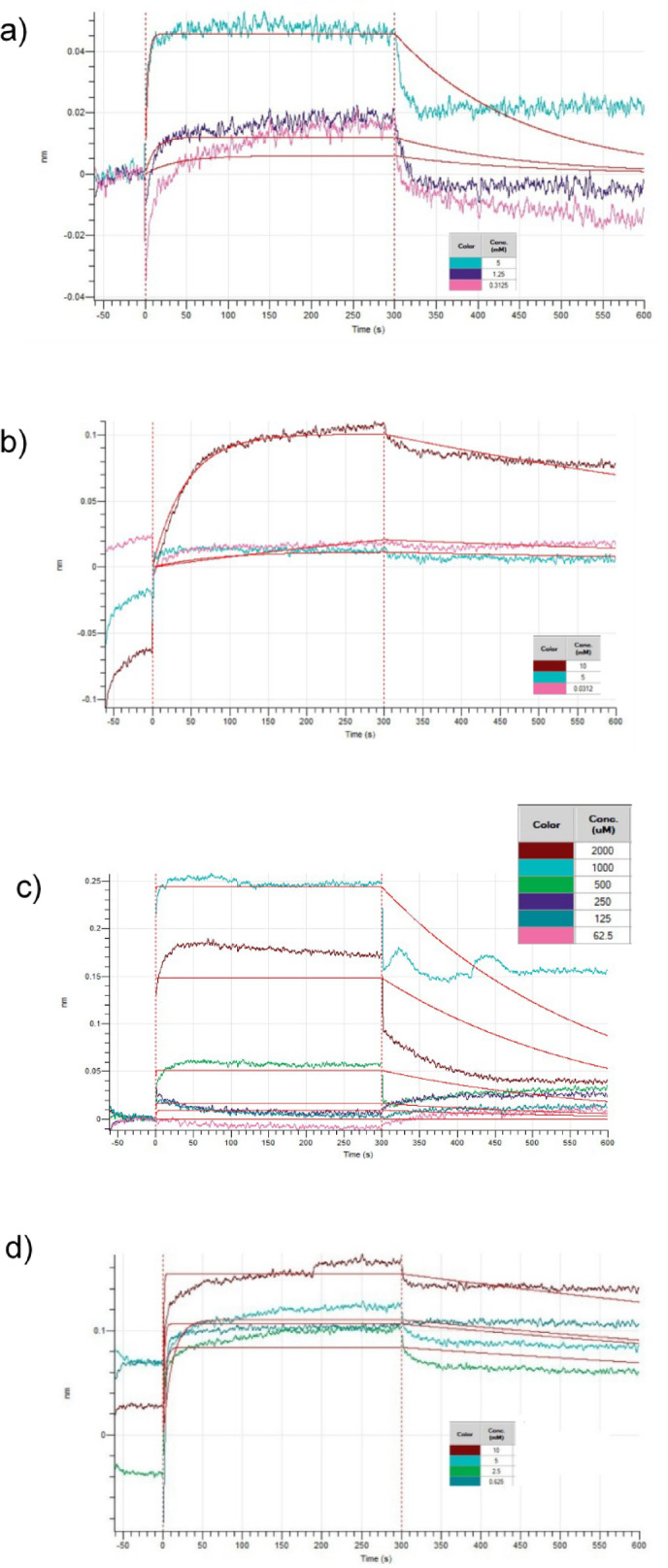
BLI sensorgrams of **(a)** menthol, **(b)** nicotine, **(c)** capsaicin and **(d)** acrolein on Human ACE2 protein. Association occurs from 0 to 300 s, and dissociation occurs from 300 to 600 s.

The binding affinities of the selected compounds on ACE2 were experimentally validated using BLI, which is presented in Table 5; Fig. 7. Both nicotine and menthol demonstrated millimolar-level binding to ACE2, while acrolein showed micromolar-level binding. The binding of these compounds was significantly weaker compared to the nanomolar level binding demonstrated by known ACE2 inhibitor MLN4760^81,82^, which also agrees with the MM-GBSA energy analysis. This suggests that these vape juice components are unlikely to cause direct inhibition of ACE2 under physiological conditions. However, significant binding responses as observed in Fig. 7 indicate interactions with ACE2 warrant further investigation under more physiological conditions.

As observed in Table 5; Fig. 7d, acrolein showed very fast association, but also dissociated quickly (high dissociation rate/k_d_), indicating that while it quickly interacts with ACE2, the binding is not stable. Therefore, despite the micromolar-level affinity constant, the binding of acrolein to ACE2 is transient and unlikely to impact ACE2 function. This behavior of acrolein is also consistent with the MD simulations, where it quickly dissociated from the binding site. In contrast, nicotine showed slow association and slow dissociation (Fig. 7b), indicating more stable binding to ACE2. This agrees with the MD simulation results, where nicotine demonstrated a negative binding free energy. While menthol and capsaicin showed a higher K_D_ value than either of those compounds, they demonstrated slower dissociation compared to acrolein. Capsaicin also demonstrated less consistent binding behavior compared to other compounds, characterized by high noise levels and higher variability across replicates. The relatively low affinity of menthol and capsaicin agrees with the MM-GBSA calculations, suggesting the hydrophobic nature of those two compounds may make the binding unfavorable in aqueous conditions.

Overall, these results indicate that nicotine forms moderately stable interactions with ACE2, acrolein binds quickly but unstably, and menthol and capsaicin occupy an intermediate position in both binding rate and stability. However, since this BLI was conducted in an aqueous buffer system which is different from the complex environment in the human respiratory tract, the binding parameters should be interpreted as molecular-level interactions rather than physiological impacts.

**Table 5:** Association rate (K_a_), dissociation rate (K_d_) and affinity constant (K_D_) of menthol, nicotine, capsaicin and acrolein on ACE2 receptor. All values are expressed as mean ± standard deviation based on three independent replicates.

Compound	K_a_ (1/Ms)	K_d_ (1/s)	K_D_ (M)
Menthol	(6.061 ± 5.366) × 10^1^	(1.051 ± 0.383) × 10^− 2^	(0.817 ± 1.218) × 10^− 3^
Nicotine	(1.857 ± 2.898) × 10^1^	(7.155 ± 5.393) × 10^− 4^	(2.208 ± 2.898) × 10^− 4^
Capsaicin	(2.205 ± 3.772) × 10^3^	(1.447 ± 2.472) × 10^− 1^	(2.889 ± 4.909) × 10^− 3^
Acrolein	(1.196 ± 2.038) × 10^5^	(0.660 ± 1.109) × 10^− 1^	(2.069 ± 2.375) × 10^− 6^

## Conclusions

This study presents a computational and experimental analysis of how common vape juice components including nicotine, menthol, and capsaicin, interact with human ACE2 protein, a critical regulator of the renin–angiotensin system and entry receptor for SARS-CoV-2. Molecular docking and molecular dynamics simulations demonstrated that menthol and capsaicin maintained stable binding to the active site of ACE2. In comparison, the binding of formaldehyde and acrolein was highly unstable. While nicotine exhibited some initial instability, it also relocated to a stable position within the binding pocket. Trajectory-based MM-GBSA analyses suggested nicotine as the most thermodynamically favorable ligand, while menthol and capsaicin also demonstrated strong non-covalent interactions. Biolayer interferometry confirmed these findings by showing that nicotine engaged in moderately stable binding with ACE2, as evidenced by slow dissociation rate.

Together, these results suggest that vape constituents can interact with the active site of ACE2, warranting further investigation of potential functional implications. However, the impact of vape components binding on ACE2 function and any possible consequences on systems biology remain uninvestigated and require in-vitro and in-vivo validation. Additionally, while the present molecular dynamics analyses provide insight into ligand–ACE2 interactions, they are based on a single simulation trajectory per ligand, and additional replicate simulations will be valuable for strengthening statistical reliability in the observed trends. Directions for future work include determining whether these interactions impact ACE2 enzymatic activity or affect downstream signaling or viral protein binding. Validating these interactions and their potential physiological impacts in in-vivo models, elucidating the synergistic or antagonistic effects of these compounds under physiological conditions, and evaluating how these chemicals interact with other receptor systems such as TRP channels and nAChRs in an integrated network can help obtain a comprehensive understanding of the impact of vaping on human biology.

## Supplementary Information

Below is the link to the electronic supplementary material.

Supplementary Material 1

## Data Availability

All relevant data are included in the manuscript or supplementary data. Any additional information is available from authors upon request.
