# Supplementary material for: Evaluation of molecular interactions of vaping juice components with ACE2 receptor
Source: Sci Rep. 2026 Feb 21;16:10118. doi: 10.1038/s41598-026-39533-0 (PMC13022229; doi:10.1038/s41598-026-39533-0)
Supplement: Supplementary file 1 — Supplementary Material 1 [file 41598_2026_39533_MOESM1_ESM.docx]

**Supplementary Data**

**Sequence of ACE2**

The sequence of ACE2 monomeric unit which was used for molecular docking and MD simulations (obtained from RCSB entry 3D0G)

STTEELAKTFLETFNYEAQELSYQSSVASWNYNTNITEENVQNMNNAGDKWSAFLKEQSTLAQMYPLQEIQNLTVKLQLQALQQNGSSVLSEDKSKRLNTILNTMSTIYSTGKVCNPDNPQECLLLEPGLNEIMANSLDYNERLWAWESWRSEVGKQLRPLYEEYVVLKNEMARANHYEDYGDYWRGDYEVNGVDGYDYSRGQLIEDVEHTFEEIKPLYEHLHAYVRAKLMNAYPSYISPIGCLPAHLLGDMWGRFWTNLYSLTVPFGQKPNIDVTDAMVDQAWDAQRIFKEAEKFFVSVGLPNMTQGFWENSMLTDPGNVQKAVCHPTAWDLGKGDFRILMCTKVTMDDFLTAHHEMGHIQYDMAYAAQPFLLRNGANEGFHEAVGEIMSLSAATPKHLKSIGLLSPDFQEDNETEINFLLKQALTIVGTLPFTYMLEKWRWMVFKGEIPKDQWMKKWWEMKREIVGVVEPVPHDETYCDPASLFHVSNDYSFIRYYTRTLYQFQFQEALCQAAKHEGPLHKCDISNSTEAGQKLFNMLRLGKSEPWTLALENVVGAKNMNVRPLLNYFEPLFTWLKDQNKNSFVGWSTDWSPYAD

**Table S1 – Distances between Zn^2+^ ion and coordinating atoms in each ligand**

| **Ligand** | **Distance to Zn^2+^ ion** |
| --- | --- |
| Menthol | 2.16 |
| Nicotine | 2.23 |
| Capsaicin | 2.02 |
| Formaldehyde | 2.17 |
| Propylene glycol | 2.05 and 2.07 |
| Glycerol | 2.12 and 2.20 |
| Acrolein | 2.13 |
| MLN4760 (control) | 2.15 |

**Table S2 – Interaction entropies (TΔS) values for the binding of the vape chemical components on ACE2**

| Molecule | Interaction entropy (TΔS) (kcal/mol) | Average ΔG bind with entropy correction |
| --- | --- | --- |
| Menthol | -4.420 | 15.418 |
| Nicotine | -7.758 | -17.504 |
| Capsaicin | -7.011 | 3.330 |
| MLN-4760 | -21.210 | -47.551 |


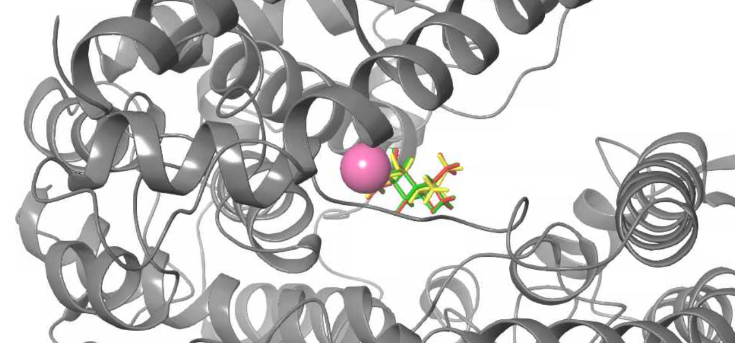

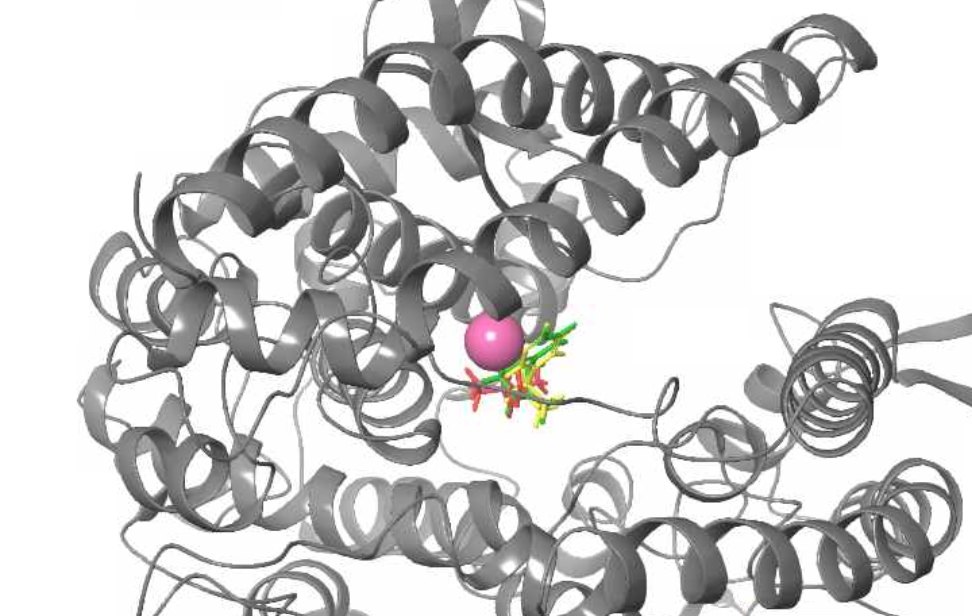


a)

b)

c)

d)


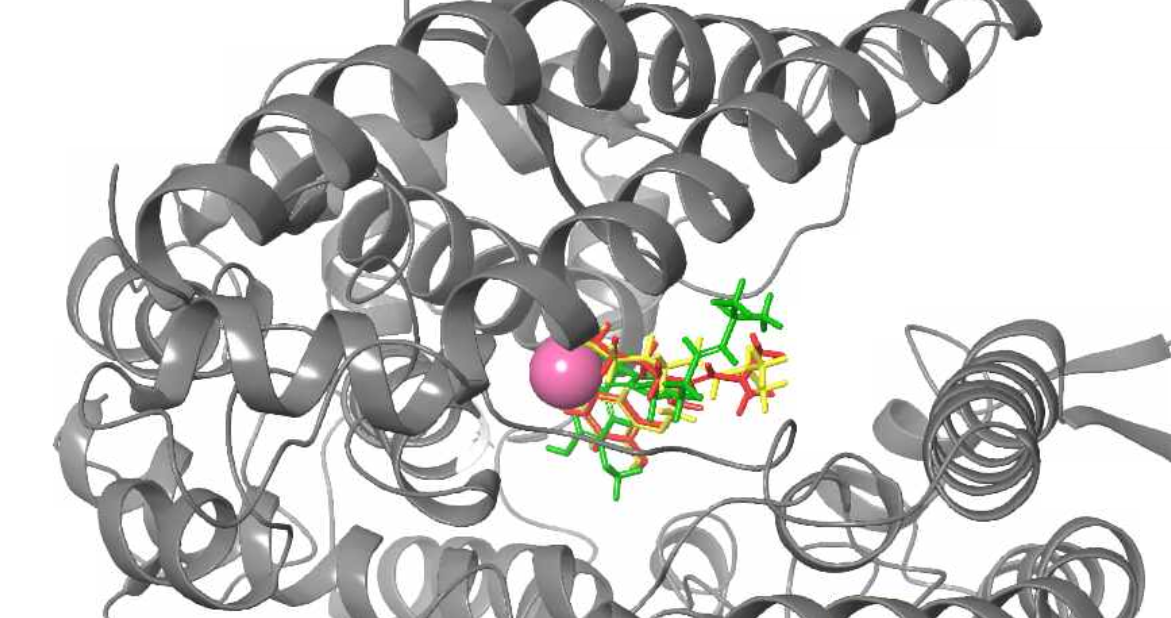

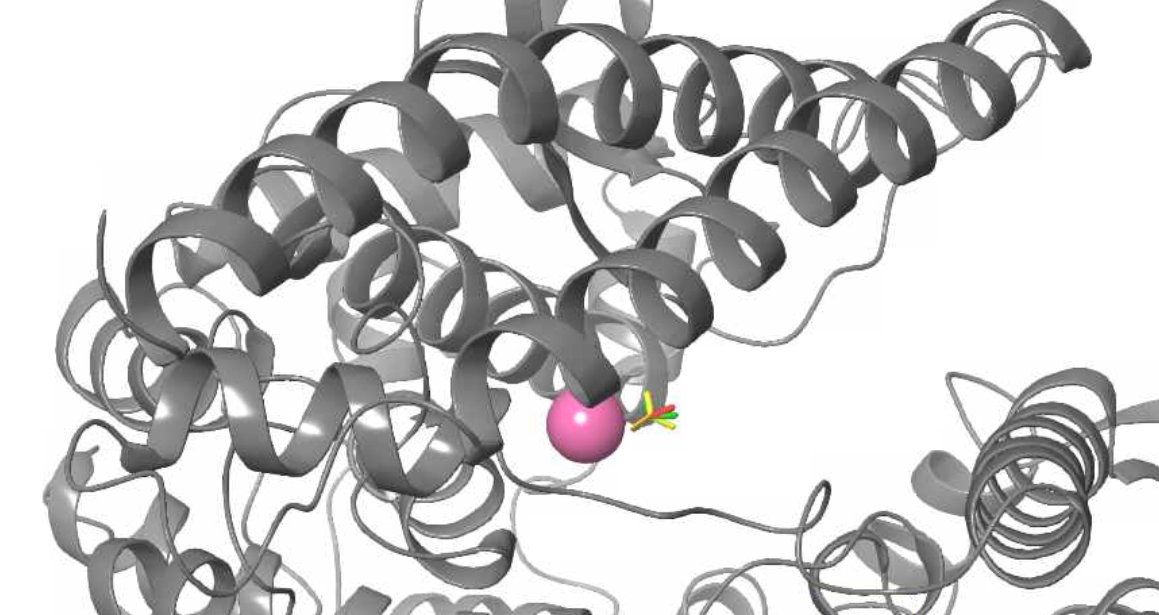


e)

f)


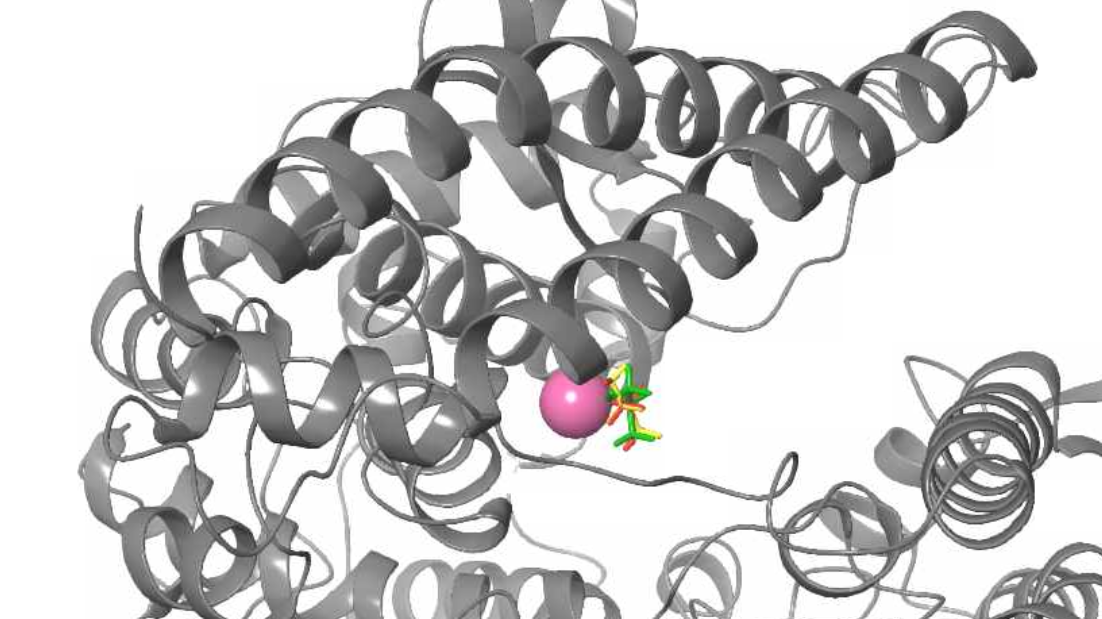

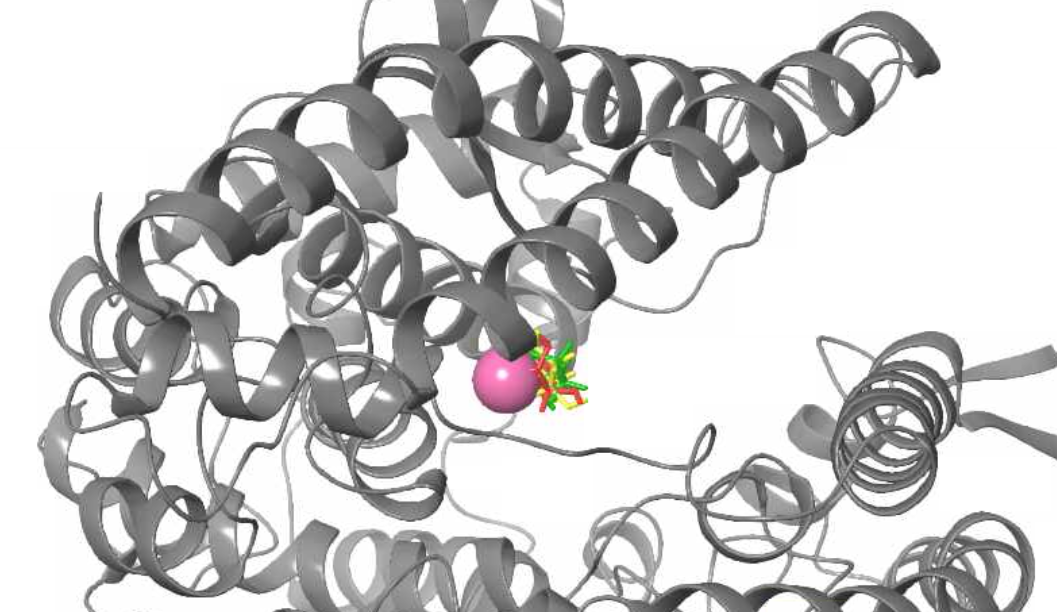

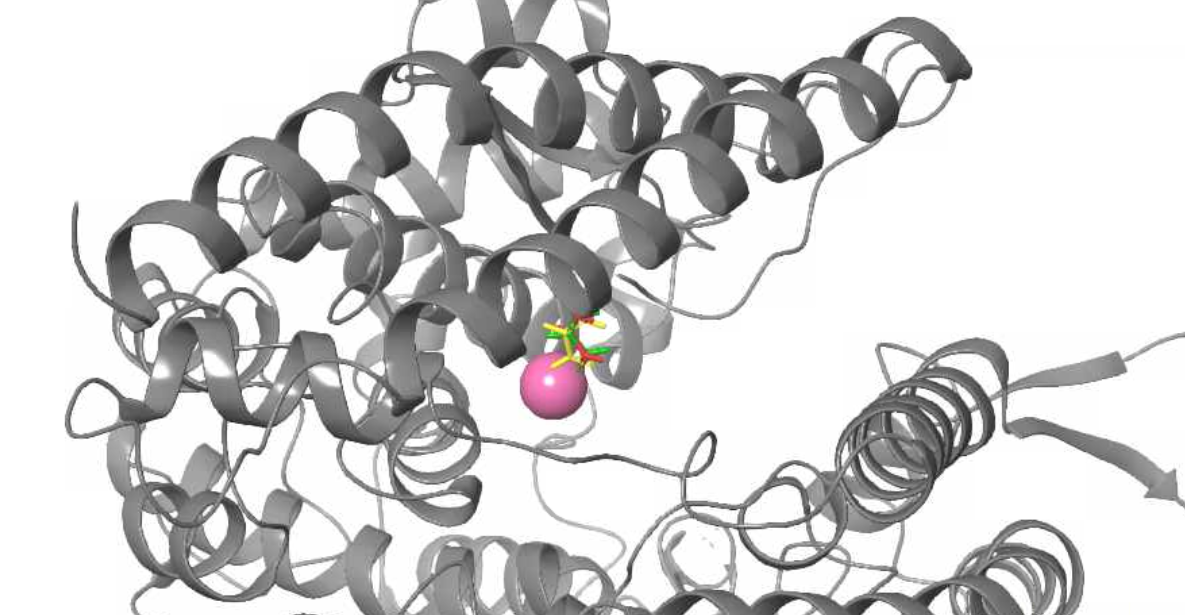

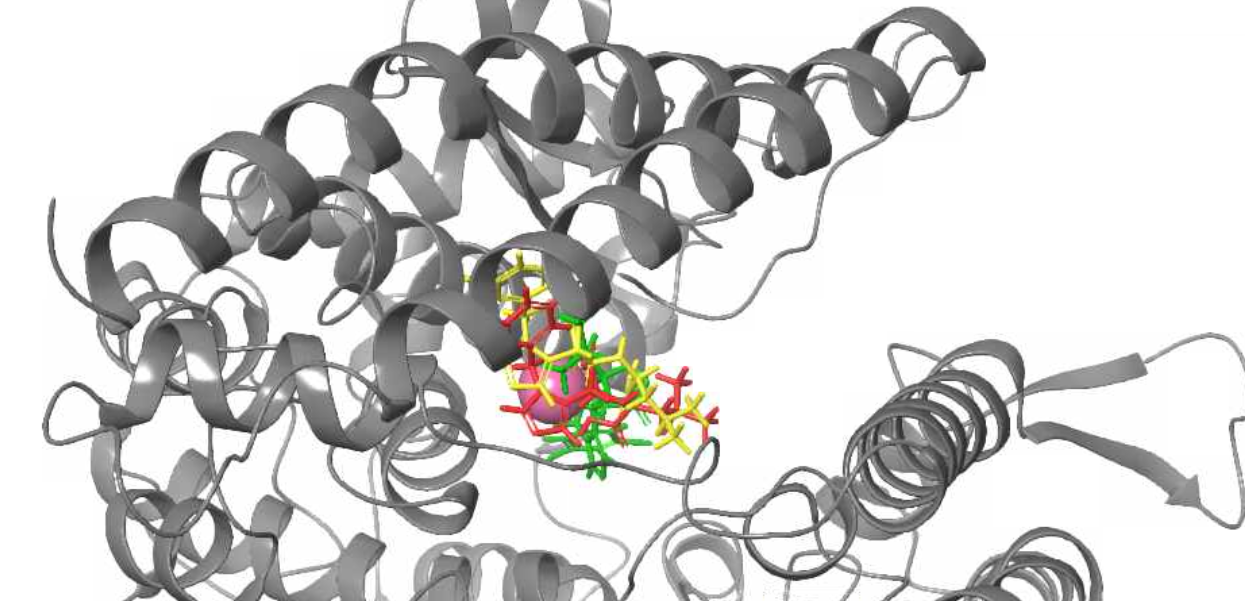


g)

h)

**Figure S1. The clustering of top three binding conformations for a) menthol, b) nicotine, c) capsaicin, d) formaldehyde, e) propylene glycol, f) glycerol, g) acrolein and h) MLN4760 at the binding site. The top three conformations of each molecule are shown in green, yellow and red, while the Zn^2+^ ion is shown as a pink sphere.**

a)


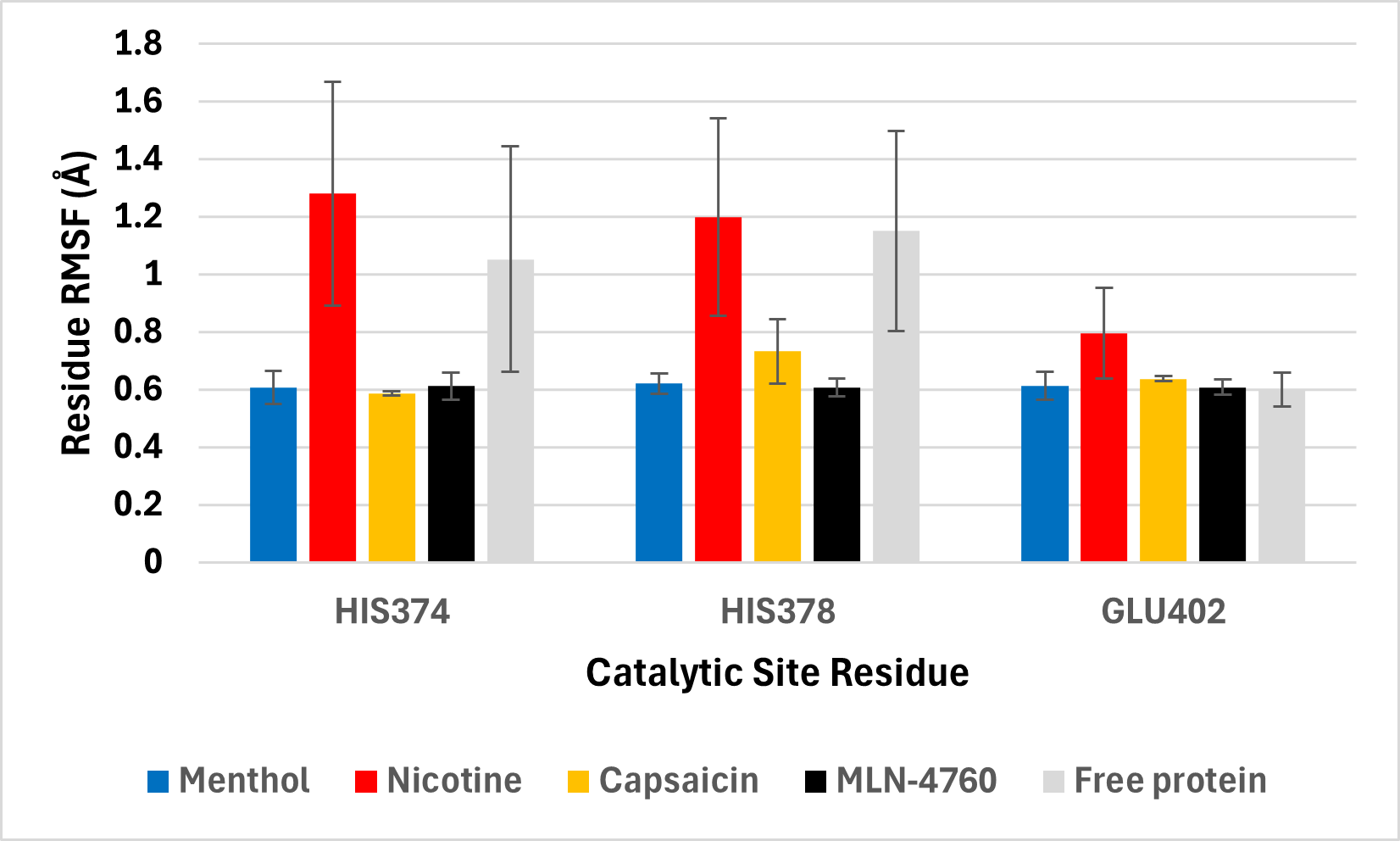

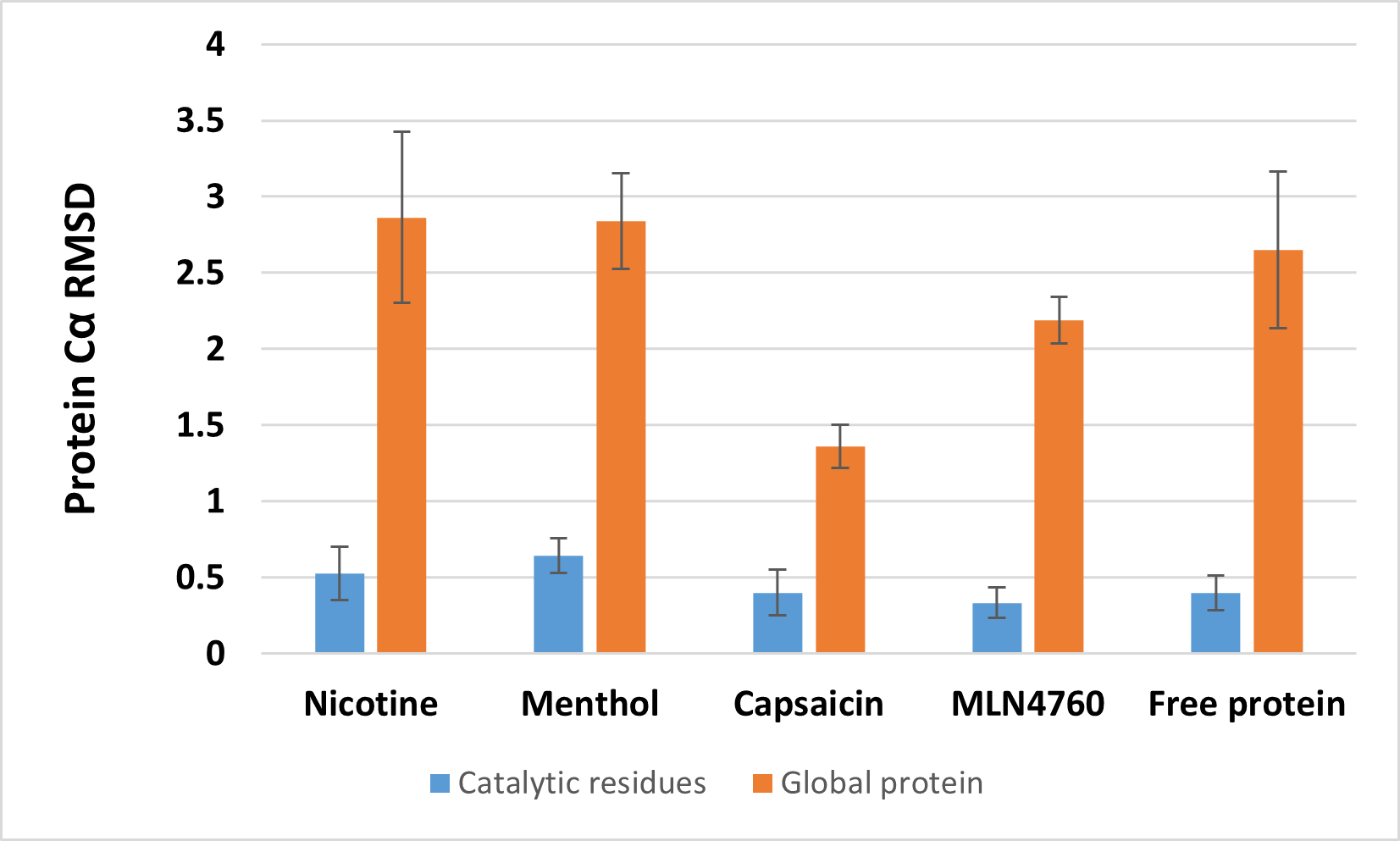


b)

*

*

*

*

*

*

**Figure S2. a) RMSF of the catalytic site residues HIS374, HIS378 and GLU402 of protein-ligand complexes, b) Global protein and catalytic site Cα RMSD for protein-ligand complexes. The instances which were statistically significant compared to the free protein are marked with an asterisk *.**


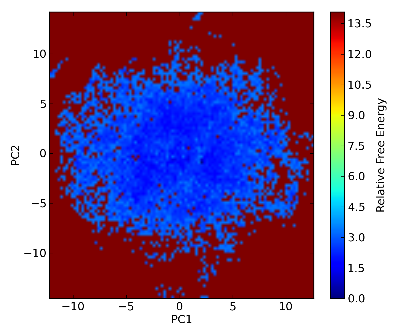

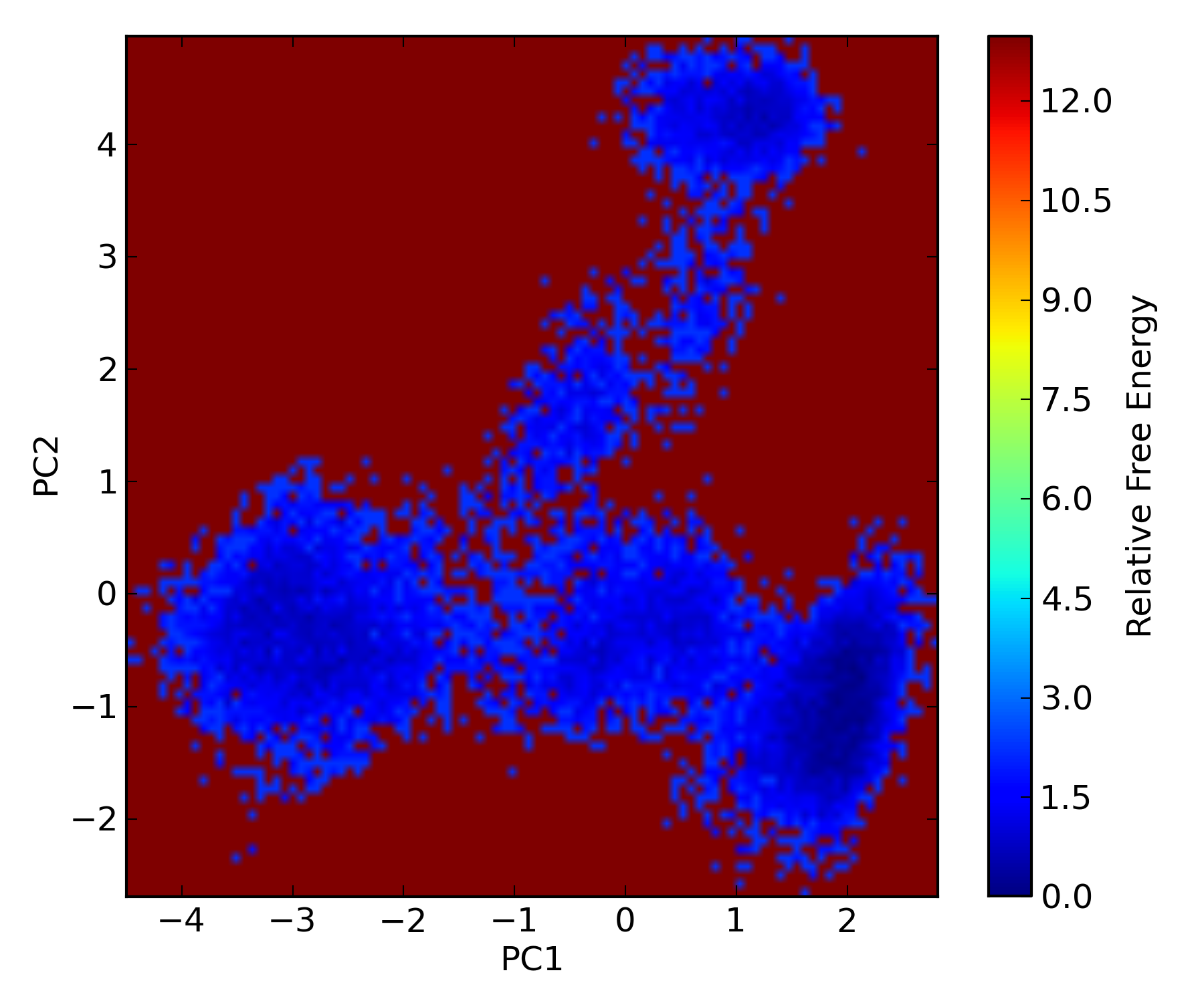

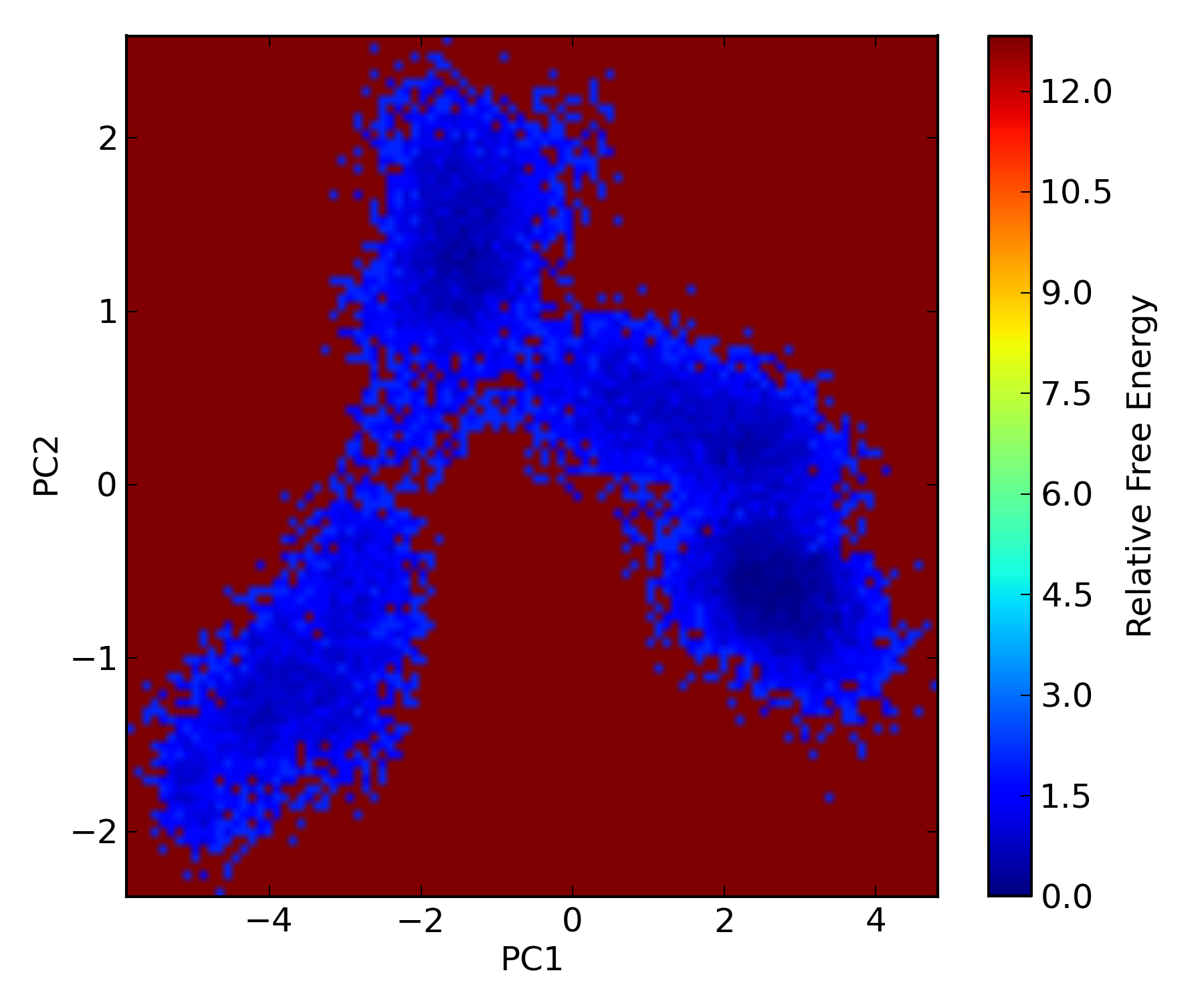


Free protein

Menthol

Nicotine


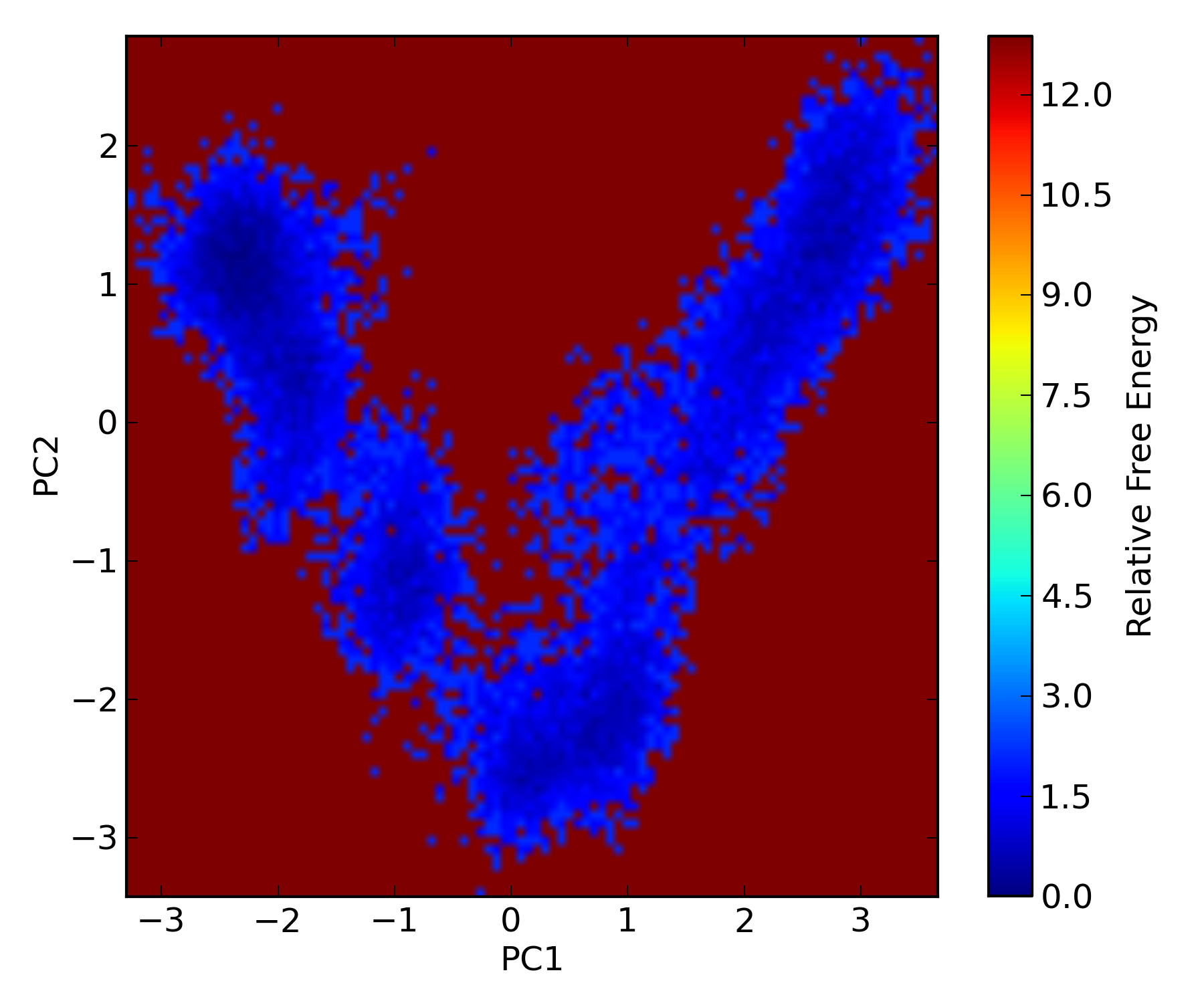

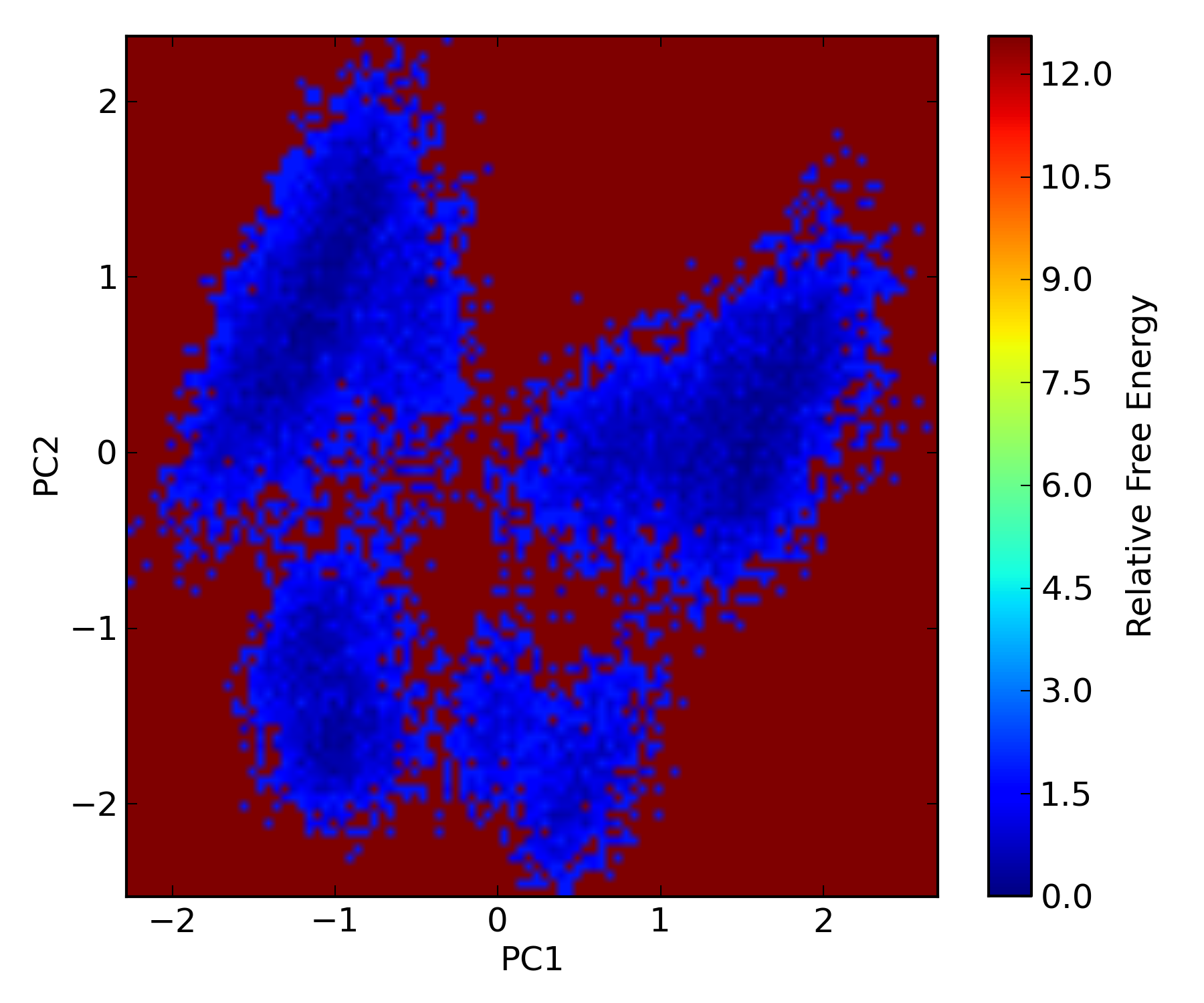


Capsaicin

MLN4760

**Figure S3 – Free energy landscape plots for free ACE2 protein, and ACE2 protein complexed with menthol, nicotine, capsaicin and MLN4760**

**Script used to conduct principal component analysis**

import mdtraj as md

import numpy as np

import pandas as pd

# ---- Load trajectory ----

traj = md.load('protein.xtc', top='protein.pdb')

# ---- Select CA atoms and align ----

ca = traj.atom_slice(traj.topology.select("protein and name CA"))

ca = ca.superpose(ca, frame=0) # important: assign back

# ---- Build feature matrix (frames x coordinates) ----

X = ca.xyz.reshape(ca.n_frames, -1)

# ---- Center data ----

X_centered = X - X.mean(axis=0)

# ---- PCA via SVD ----

U, S, Vt = np.linalg.svd(X_centered, full_matrices=False)

# ---- Projected coordinates ----

proj = U * S

# ---- Variance explained ----

variance = (S**2) / np.sum(S**2)

print(variance[:5])

# ---- Extract first two PCs ----

pc1 = proj[:, 0]

pc2 = proj[:, 1]

# ---- Save projections ----

pd.DataFrame({'PC1': pc1, 'PC2': pc2}).to_csv("PCA_projection.csv", index=False)

**Trajectory videos for MD simulations**

The trajectory videos for the molecular dynamics simulations can be found at the following Zenodo repository

https://zenodo.org/records/17643515
